# On urban maladaptation in times of epidemics

**DOI:** 10.1038/s41598-025-33158-5

**Published:** 2026-01-06

**Authors:** Mikhail Sirenko, Alexander Verbraeck, Tina Comes

**Affiliations:** Faculty of Technology, Policy and Management, Delft University of Technology, Delft, Netherlands

**Keywords:** Urban, Adaptation, Maladaptation, Simulation, Behaviour, Epidemic, Climate-change adaptation, Natural hazards

## Abstract

Epidemics are long-lasting and transboundary crises that challenge traditional approaches. Given the complexity and interconnectedness of modern cities, interventions can lead to unintended consequences or maladaptation. Although adaptation is central to resilience, crisis management often focuses on short-term response, leaving a gap in understanding urban adaptation and maladaptation. This study examines the impacts of uniform interventions across diverse urban districts to assess this (mal)adaptive process. We use the COVID-19 pandemic in The Hague, Netherlands, as a case study, employing a large-scale agent-based model. We find that without an intervention, the high-contact city centre becomes an infection hotspot due to the transient population it attracts. Conversely, the outer residential district, with fewer amenities, experiences infections primarily among its residents. A uniform lockdown policy significantly reduces infections in the city centre by limiting mobility and social interactions, but inadvertently increases risk in the outer residential district. Using the Urban Adaptation Index (UAI), we demonstrate that these uncontextualised policies can constitute maladaptation, confirming the unintended consequences of ’one-size-fits-all’ approaches. Our results underscore this need, leading us to propose an updated, equity-oriented crisis management framework that accounts for the heterogeneous nature of modern cities.

## Introduction

Driven by factors like climate change and biodiversity loss, epidemics are expected to take an increasing toll in the coming years^1^. These events often become long-term, transboundary crises^2^ with modern cities as their centres of gravity. However, cities are not uniform; they are increasingly heterogeneous systems^3^. Due to segregation and socioeconomic inequalities, two adjacent districts can be vastly different^4^. This heterogeneity, which concentrates vulnerable populations with pre-existing health conditions or lower incomes^5,6^, fundamentally challenges traditional crisis management, which has historically focused on short-term, sector-specific events.

In response to crises like the COVID-19 pandemic, governments often implement uniform, country-scale interventions, such as lockdowns. While appearing fair by applying the same rules to all, these ’one-size-fits-all’ strategies often ignore the diverse urban and social fabrics that shape behaviour and vulnerability^7,8^. This can lead to unintended consequences, or *maladaptation* - a concept borrowed from climate change literature referring to interventions that inadvertently worsen the situation or increase vulnerability for certain groups^9,10^. Instead of fostering adaptation, a uniform policy may shift risk, amplifying inequalities rather than mitigating them^11^.

Understanding *how* and *why* such maladaptation occurs at the urban scale requires appropriate methods, but current approaches face a methodological gap. Global or national-level studies offer broad comparisons but lack the high spatial granularity needed for effective local action^12,13^. Conversely, local case studies provide essential context but struggle with generalisability^14^. To bridge this gap, computational models are needed - specifically, large-scale spatially explicit simulation models that move beyond national averages^15–18^. Because virus spread is driven by human mobility and contact behaviours^19,20^, models must be centred on this behaviour at a high spatial and temporal resolution to be able to analyse the complex, emergent impacts of interventions^21,22^.

This study follows an exploratory approach^23,24^ to develop a framework for identifying and measuring urban maladaptation during an epidemic. Instead of seeking predictions, our work aims to build the theoretical and conceptual foundations for understanding the mechanisms that drive these unintended outcomes. To do this, our research is threefold. First, we develop a large-scale agent-based model (ABM), a full-scale ’digital replica’ of a city, to serve as a ’computational laboratory’ for testing intervention scenarios. Second, we conceptualise how maladaptation, as defined by^9^, unfolds in response to an urban crisis, focusing on citizen adaptive behaviour and supplementing it with a simple and interpretable Urban Adaptation Index (UAI) to quantify how uniform policies spatially redistribute risk across diverse districts. We apply this framework to the COVID-19 pandemic in The Hague, Netherlands. Finally, we propose an updated adaptation and equity-oriented crisis management cycle.

## Results

### Central vs outer residential: equal access but unequal distribution of essential services

**Fig. 1:**
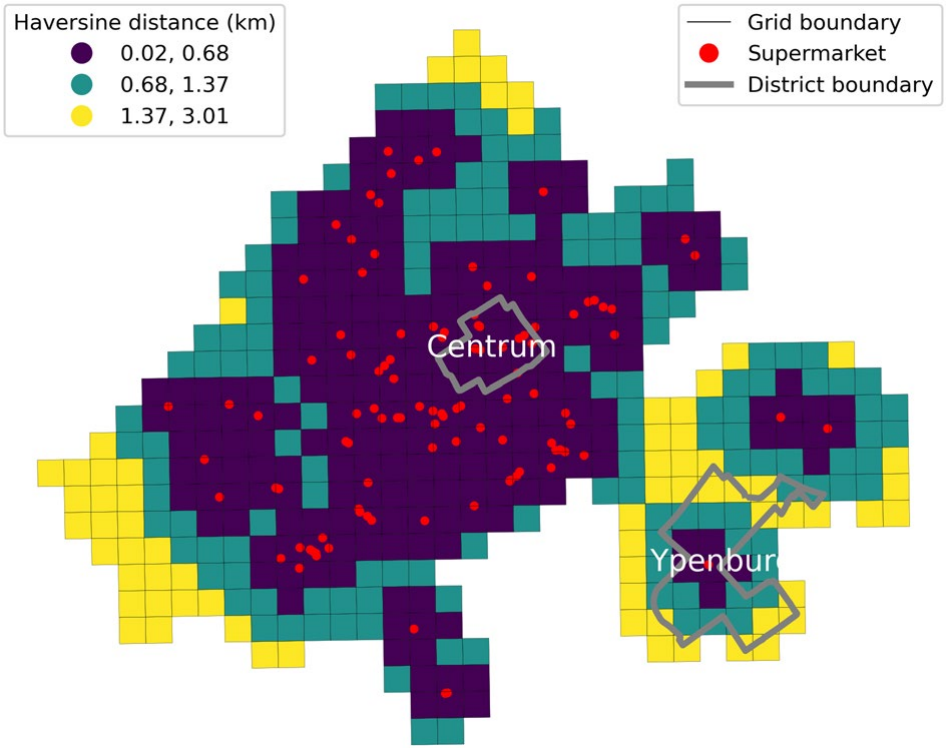
Supermarket proximity in Central and Outer residential districts. Red dots mark supermarkets; district boundaries are grey; grid cells are colour-shaded by Haversine distance to the nearest supermarket (shorter = closer). Black grid lines indicate analysis cells.

The Hague, the third most populous city in the Netherlands, presents a diverse urban landscape with varying patterns of service distribution and citizen behaviour across its districts. Our analysis focuses on two exemplar but distinct districts (Fig. 1): Centrum, located in the city centre (further Central), and Ypenburg, an affluent Outer residential district (further Outer residential). Despite having comparable population sizes and areas, these districts exhibit different urban and social characteristics.

We find that the Central district, with a population of 26,833 residents over two km$^{2}$, and the Outer residential district, with 21,643 residents over five km2, both have relatively high access to essential services such as grocery shops. The median distance to the closest supermarket is 0.3 km for the Central and 1.1 km for the Outer residential district. Central has a higher percentage of elderly residents, a significant proportion of individuals with chronic health conditions, and a larger segment with low income and low educational attainment. Conversely, the Outer residential district has a population with fewer chronic health conditions and psychological distress, higher income, and higher educational attainment.

The Hague’s Central district serves as a hub of activity, hosting a significant proportion of the city’s amenities. With 2,440 amenities (2019 data; 17% of the city’s total), Central has 113 amenities per 1,000 residents, significantly higher than Outer residential with 292 amenities (2% of the total) or 11 per 1,000 residents. This uneven distribution extends to essential services such as supermarkets, where Central has ten compared to the Outer residential district with just one. This centralisation of services in the Central district is arguably designed to cater not only to its residents but also to the transient population, visitors, and tourists passing through the city centre.

**Fig. 2:**
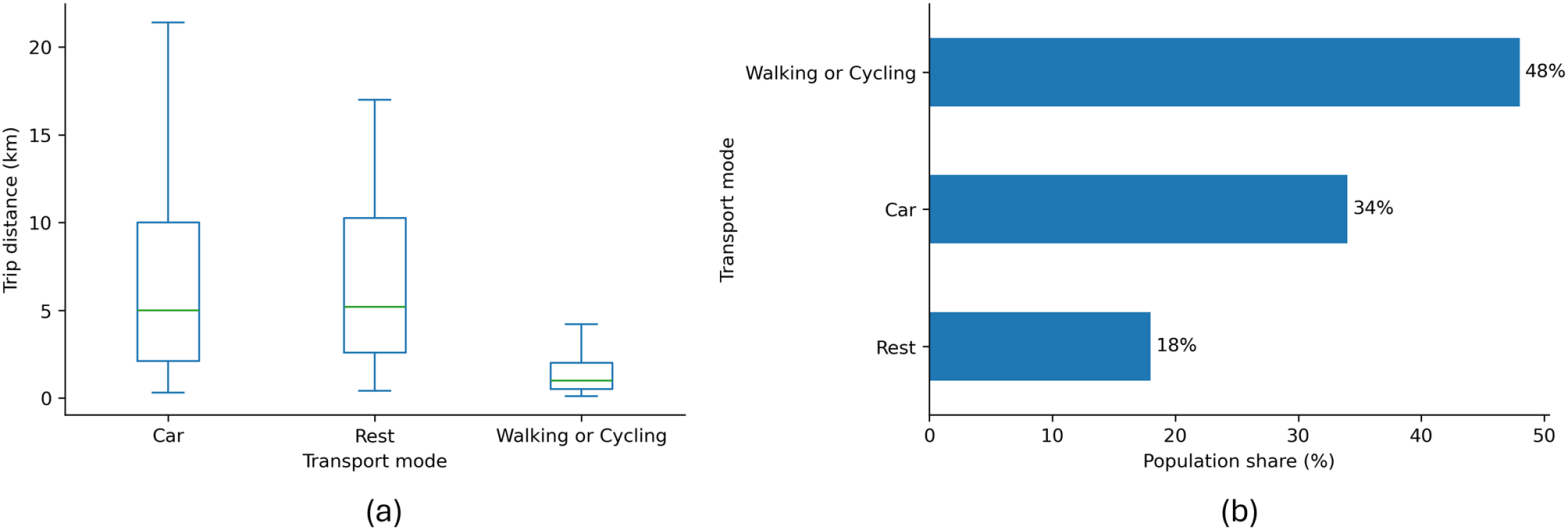
Grocery trips: distance by mode and population share (pre-pandemic baseline). (**a**) Trip-distance distributions by transport mode; boxes show median, interquartile range, and whiskers at 1.5$\times$IQR. (**b**) Share of residents by preferred transport mode for grocery shopping.

Previous research has found that the behaviour of citizens in terms of transportation and shopping habits is an important driver of the spread of a virus^25,26^. From Fig. 2, we see that before the pandemic, a significant proportion of grocery shopping trips by residents of highly urbanised areas in the Netherlands were made by bicycle or on foot (49%), with the remainder split between car (37%) and public transport (13%). The median distances travelled for groceries are 0.6 km on foot, 1.5 km by bicycle, and 5 km by car. These distances indicate that most shopping activities occur within the area where the resident is: either at home, work, or recreation, forming trip-chaining.

**Fig. 3:**
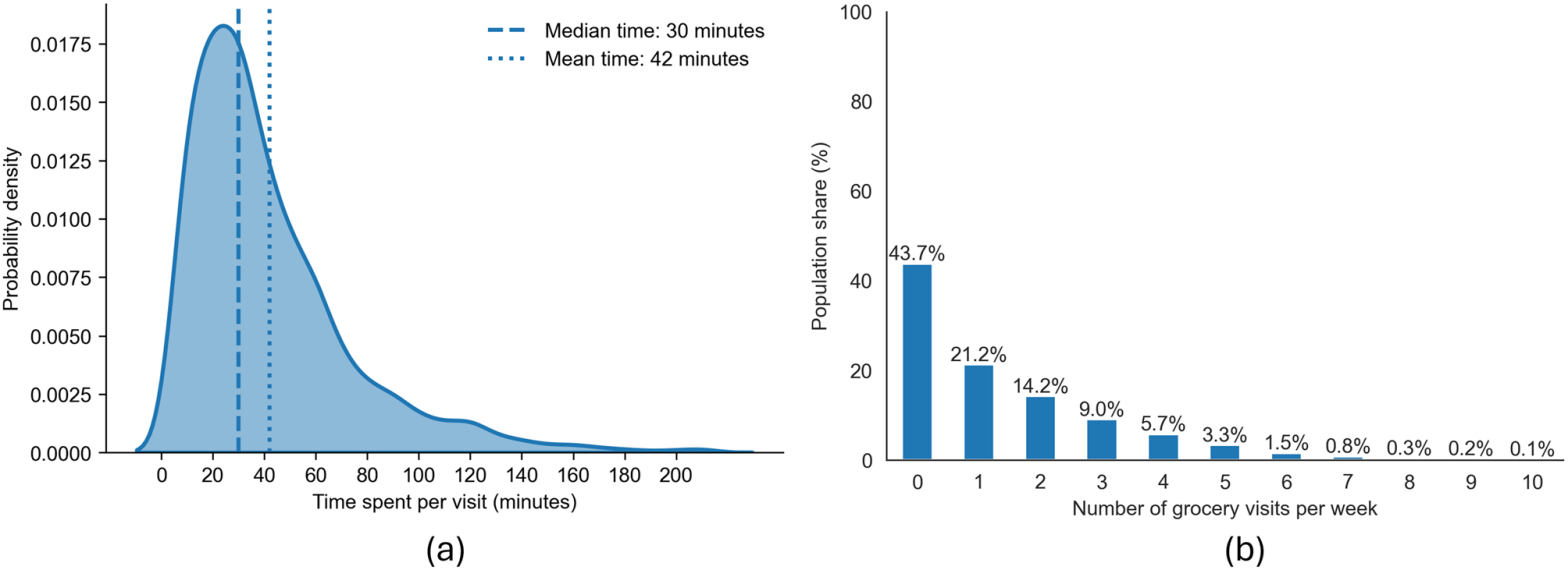
Grocery shopping behaviour (pre-pandemic baseline). (**a**) Probability density of per-visit duration: median 30 minutes and mean 42 minutes. (**b**) Weekly visit frequency as population shares.

Another important aspect of the virus spread is how much time is spent with potentially infected individuals in indoor spaces^27^. We can see from Fig. 3 that the median time spent on grocery shopping is 30 minutes, typically involving one visit per week (44%).

Given the two districts’ very different urban and social fabric, we hypothesise that the adaptation to government interventions during COVID-19 will also differ. Understanding adaptive citizen behaviour in these districts is key to analysing potential maladaptation.

### The city centre is a hotspot under the no-response scenario

**Fig. 4:**
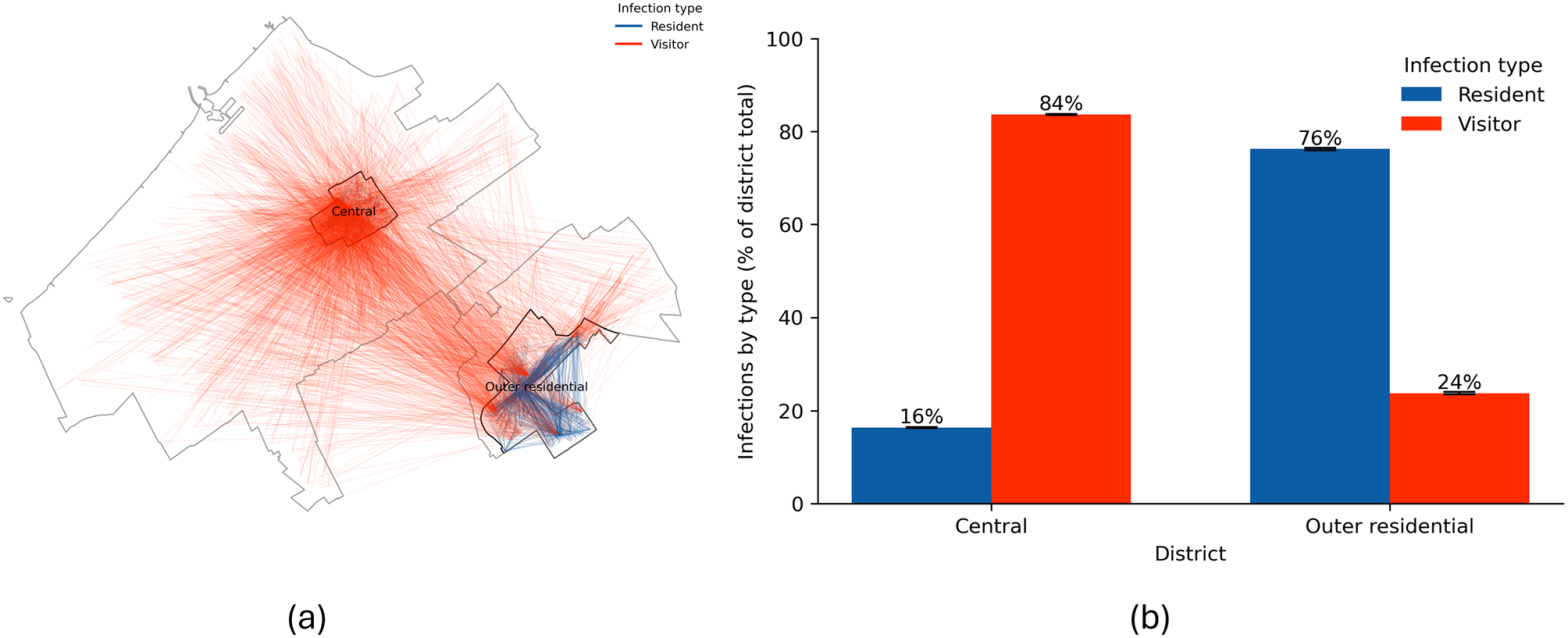
Impact of the No-response on infection spread. (**a**) Snapshot on day 25 of one simulation: lines connect an infected agent’s residence to the location of infection; red = *visitor infections* (infected outside home district), blue = *resident infections*. (**b**) Mean proportion of infections by agent type within each district at the end of the simulation, averaged across ten runs; error bars indicate ±1 SD. Percentages are relative to total infections within each district.

Few countries worldwide have approached the COVID-19 pandemic without imposing any restrictions on their populations^28,29^. Figure 4 visualises the potential consequences of such a no-restriction approach across the urban districts of The Hague. In this scenario, the city does not implement any measures to control the spread of the virus. Thus, we assume that citizens would continue their daily routines, businesses and services remain open, and other activities remain unchanged. This ’business-as-usual’ approach leads to a rapid spread of the virus (see Supplementary Information for the temporal progression of the virus). In both districts, the spread peaks at around 25 days. Following this peak, the number of infections declines as most of the population is infected and subsequently recovers. Panel a in Fig. 4 captures a snapshot on day 25 of a single simulation run. This visualisation uses lines to connect the residences of agents with the locations where they contracted the virus. Red lines indicate that the infected agent was a visitor to the district, while blue lines denote infections occurring within the agent’s district of residence. We focus on displaying infections happening only in these districts of interest. However, as can be seen from the map, the visitors come from all over the city.

Despite the similar number of residents in both districts (26,833 residents in Central, and 21,643 in Outer residential on 1 January 2020,) the patterns of virus spread exhibit striking differences. The Central district, characterised by its numerous amenities and attractions, becomes an infection hub and ranks as the first across all the districts by the number of infections (mean of 38,772 total infections or 8.19% of all infections across ten simulation model runs, see Supplementary Information for the complete overview of ranking). People from across the city travel to the Central district for work, leisure, and shopping. During these visits, some individuals contract the virus and then return to their home districts, potentially spreading the infection to household members.

Panel b demonstrates an almost opposite cumulative trend in the proportion of infections in the two study districts at the end of the model run. In the Central district, most infections (84%) occur among visitors, further driving the spread of the virus in their home districts, while only 16% of infections involve the district’s residents. This is because the Central district’s work locations, amenities, and other attractions draw numerous visitors, who then become vectors for the virus. In contrast, the Outer residential district shows a reverse trend. The Outer residential district, with fewer work locations, amenities, and attractions, is less frequently visited by outsiders. Consequently, social interactions primarily occur among residents. As a result, most infections (76%) in Outer residential are among its residents, while only 24% of infections involve visitors.

In summary, the No-response strategy makes the city centre a hotspot for the virus. From this central area, the virus spreads quickly to other districts, making it crucial to address the outbreak at its source to prevent a citywide epidemic. Additionally, the lack of a response exacerbates the vulnerability of the Central district’s residents, who are already at risk due to a combination of socioeconomic and health factors. Their increased exposure to interactions with visitors from other districts further heightens their exposure, vulnerability, and the corresponding risk of infection.

### Maladaptation and flipped risk from the city centre to the periphery

During the pandemic, the Netherlands has implemented several interventions to combat the pandemic^30^. All interventions were the same for the entire country. Only a few countries, e.g. Vietnam, tried to consider the difference in the spread of the virus at the urban and community scale, as opposed to the country scale, which resulted in district lockdowns.

**Fig. 5:**
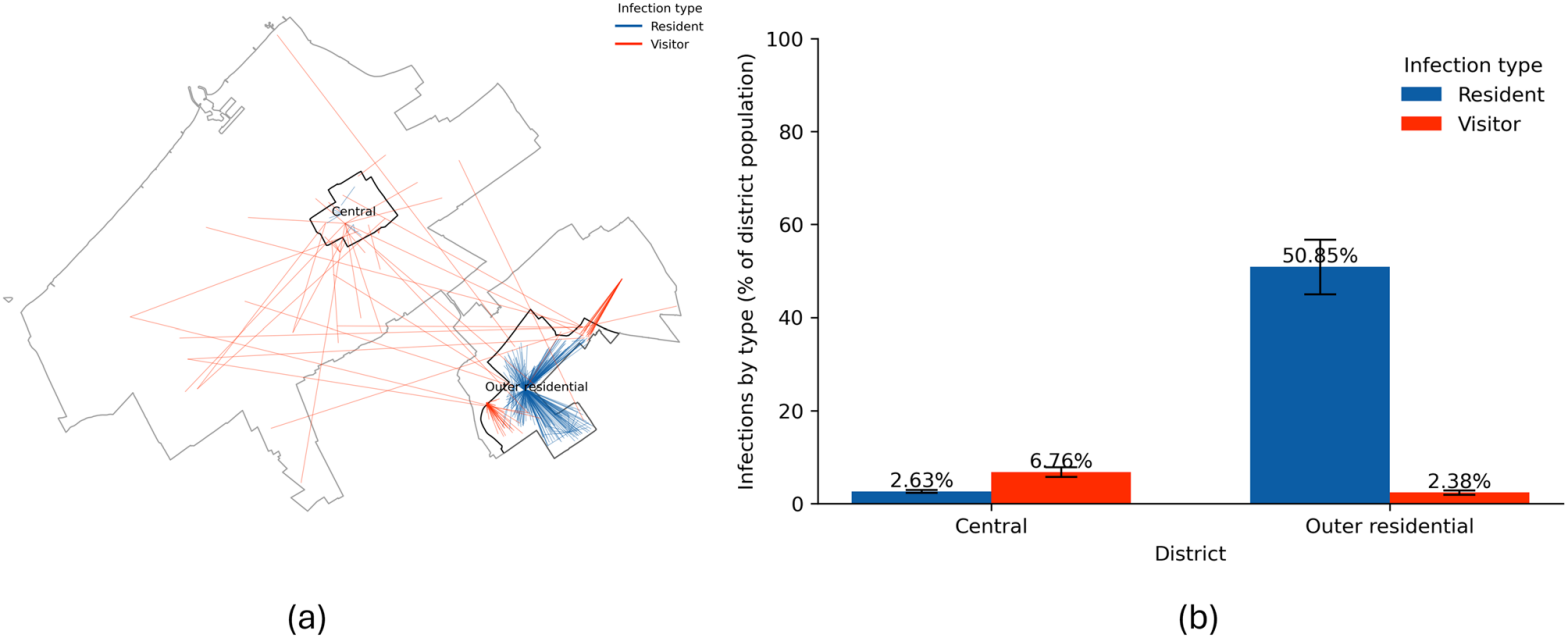
Impact of the Hard lockdown on infection spread. (**a**) Snapshot on day 25 of one simulation: lines connect an infected agent’s residence to the infection location; red = *visitor infections*, blue = *resident infections*. (**b**) Mean proportion of infections by agent type within each district at the end of the simulation, averaged across ten runs; error bars indicate ±1 SD. Percentages are relative to the total population of each district.

Figure 5 illustrates the potential impact of an extended set of measures, termed a ’Hard lockdown’. This intervention involves closing schools, childcare centres, bars, and restaurants and extending to cultural and recreational amenities and non-essential shops. Essential services such as supermarkets, hospitals, police, and fire stations remain operational. The motivation behind such a combination of measures is as follows. The Netherlands’ initial response to the pandemic did not occur immediately at the onset of the pandemic. The first COVID-19 case was reported on 27 February 2020, but stringent measures were not introduced until 15 March 2020. Following this, we activate the Hard lockdown on day 20 of the simulation model run. On this date, the government mandated the closing of schools, childcare facilities, bars, and restaurants. Despite these efforts, the infection rates continued to rise. Importantly, these policies were applied uniformly across the country without accounting for variations in vulnerability, behavioural patterns or social contact rates. Thus, by extending the types of amenities that must be closed, we wanted to explore whether these measures would slow down the spread of the virus.

The primary benefit of the Hard lockdown is a sharp reduction in the number of infected agents (see Supplementary Information for the temporal progression under the Hard lockdown). City-wide, mean total infections fell from 473,678 to 89,852 - a reduction of 383,826 (−81.0%). The upper tail decreased as well, with P90 dropping from 474,157 to 96,924 (−79.6%), indicating substantially fewer extreme outcomes and a lower burden on healthcare services.

In the Central district, our simulation shows that infections dropped significantly under the Hard lockdown compared to the No-response scenario: from 38,773 to 2,520 (−93.5%). Visitor infections declined from 32,430 to 1,815 (−94.4%), and their share of infections decreased from 83.6% to 72.0% (−11.6 pp), indicating that reducing mobility and social interactions in densely populated areas can effectively mitigate the spread of the virus.

Conversely, in the Outer residential district, where social interactions were less frequent under the No-response scenario, and the population density is lower, the simulation shows that while the number of infections decreased in the absolute sense, the reduction was less pronounced compared to the Central district: from 26,008 to 11,520 infections (a drop of 55.7%). The number of infections by the district’s visitors dropped significantly from 6,184 to 515 (91%); however, the residents’ infections decreased only by 44.5%, from 19,822 to 11,005.

In summary, the Hard lockdown, while reducing overall city-wide infections, reveals a significant maladaptation. This uniform, ’one-size-fits-all’ intervention inadvertently flips the risk profile of the city. The Central district, once the epidemic’s epicentre, benefits immensely: closing its numerous amenities and workplaces starves the virus of its primary transmission routes, causing infections to decrease by over 93%. However, this same policy is far less effective in the Outer residential district. While total infections drop here as well, the reduction is much smaller, and this district surpasses the city centre in absolute infection numbers. This outcome suggests that the lockdown, by eliminating central hubs, fails to address the different social and mobility patterns in the periphery, effectively shifting the primary infection burden from the urban core to residential areas.

**Fig. 6:**
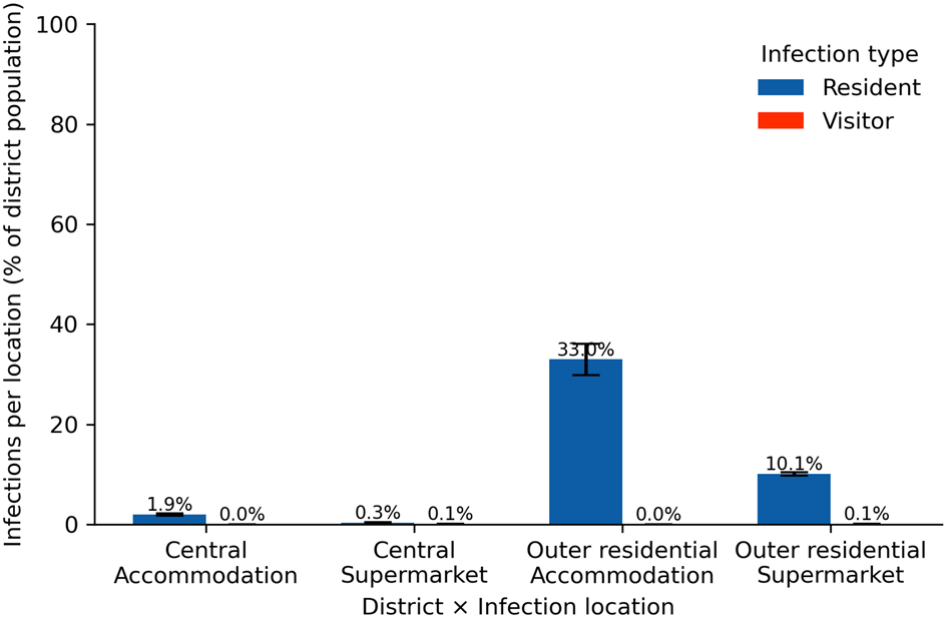
Infections by location type under Hard lockdown. Bars show the mean proportion of infections at home (*Accommodation*) or in supermarkets, split by residents (blue) versus visitors (red) and district at the end of the simulation, averaged across ten runs. Error bars indicate ±1 SD. Percentages are relative to the total population of each district.

To understand this pattern, we need to examine the role of the essential services that remained open: supermarkets. Figure 6 shows the infections by agent and location type: supermarkets versus agent homes. The figure reveals that supermarkets are pivotal in the transmission chain. Individuals adapt their behaviour and do not visit any other location anymore. Additionally, the trip chaining stops: no more shopping on the go or close to the workplaces. As a result, individuals are more likely to get infected during their supermarket visits and subsequently spread the virus within their homes. This pattern is especially pronounced in the Outer residential district, which has a higher overall number of infections compared to the Central district, indicating that supermarkets are crucial in both districts. However, due to the limited number of supermarkets serving Outer residential residents, these locations become infection hotspots. According to our findings, the Hard lockdown alters daily routines, making supermarkets the primary place where people, except for essential workers, interact with others. On the urban scale, supermarkets have served as transmission hubs for the virus, similar to airports’ role on a national and international level^31^.

### Measuring urban (mal)adaptation with an index

To assess whether a policy measure is effective or maladaptive at the urban scale, we introduce the *Urban Adaptation Index (UAI)*. The UAI is designed to capture how the **spatial distribution** of policy impacts evolves over time, specifically by tracking changes in the relative burden and ranking of different districts.

Because our simulation model generates multiple realisations (runs) for each scenario, we first aggregate infection counts across runs by computing their daily mean for each district and population group. This ensures that districts with differing numbers of valid runs are weighted equally and that the resulting trajectories represent the average expected behaviour under each policy condition.

The UAI’s key feature is its focus on the *absolute number* of infections in each district, which is then used to determine ranking. This approach allows us to track how the hierarchy of districts, based on their total infection burden, changes under a policy. This is distinct from measuring the *relative contribution* (or share) of each district to the citywide total. For each day *t* and district *d*, we take the mean daily infections $y_{b}^d(t)$ and $y_{p}^d(t)$ (calculated as described above) for both the baseline (*b*) and policy (*p*) scenarios. Districts are then ranked by this descending absolute number $r_{b}^d(t) \in \{1,.., D\}$ and $r_{p}^d(t) \in \{1,.., D\}$, where $r_{b,p}^d(t) = 1$ indicates the district with the highest number of mean infections, and *D* is the number of districts. The day-by-day change in rank quantifies how the district’s position in the urban risk hierarchy evolves under the policy:

1 $$\begin{aligned} \Delta r^d(t) = r_b^d(t) - r_p^d(t). \end{aligned}$$

A positive $\Delta r^d(t)$ indicates that the district moved *up* the ranking (toward rank 1) under the adaptive measure, signalling potential maladaptation. This means that even if the policy reduced total infections everywhere, this district’s position in the hierarchy of hotspots worsened. Conversely, a negative value indicates an improvement (the district’s rank moved down, away from rank 1).

To smooth day-to-day fluctuations, we evaluate the cumulative mean:

2 $$\begin{aligned} \overline{\Delta r}^d(t) = \frac{1}{t}\sum _{\tau =1}^{t} \Delta r^d(\tau ). \end{aligned}$$

which represents the average change in rank up to time *t*. The final value $\overline{\Delta r}^d(T)$ at the end of the evaluation window provides a summary measure of each district’s adaptive trajectory:

- $\overline{\Delta r}^d(T) < 0$: the district’s average rank improved - *adaptive response*;
- $\overline{\Delta r}^d(T) \approx 0$: the district’s average rank remained stable - *neutral response*;
- $\overline{\Delta r}^d(T)> 0$: the district climbed in the ranking - *maladaptive response*.

To illustrate, imagine a city with three districts: A, B, and C. In the ’No-response’ baseline, their infection ranking is: 1. District A (100 cases), 2. District B (50 cases), and 3. District C (10 cases). A policy is then implemented. In the ’Policy’ scenario, total infections drop, but unevenly: 1. District B (30 cases), 2. District A (20 cases), and 3. District C (5 cases). The UAI highlights this shift. District A’s rank improved from 1 to 2 (a positive adaptive outcome). However, District B’s rank worsened from 2 to 1. Even though District B saw an absolute reduction in cases (from 50 to 30), it became the city’s new number one hotspot, indicating a potential maladaptive outcome in terms of spatial equity.

It is important to note that the UAI is not intended as a standalone metric. It is designed to reveal shifts in the spatial hierarchy of risk, not to capture the overall (absolute) effectiveness of a policy. It should therefore be used alongside other metrics that measure the potential impact of a measure.

**Fig. 7:**
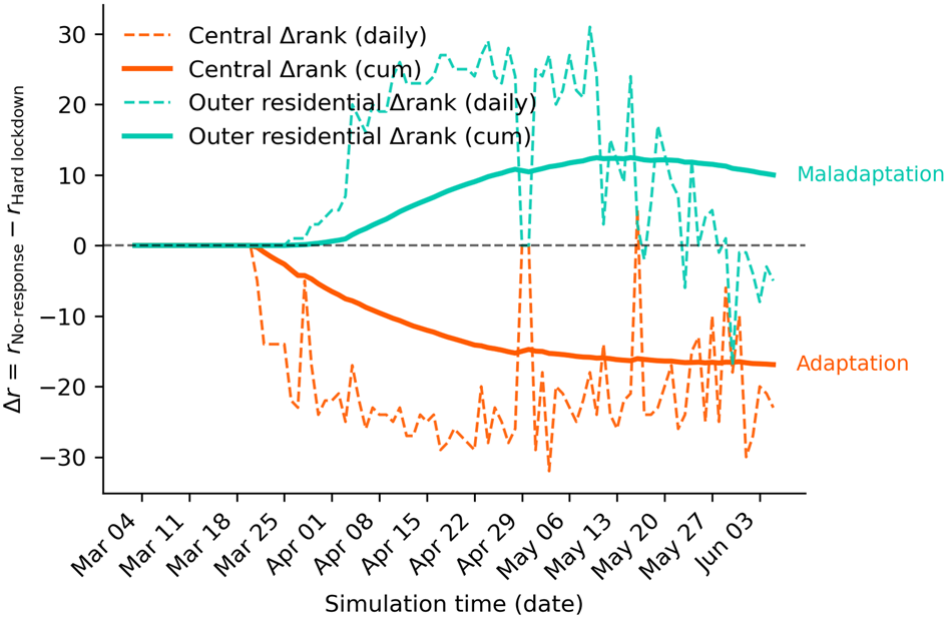
Temporal evolution of the Urban Adaptation Index (UAI) based on district rank changes under the Hard lockdown relative to the No-response scenario. dashed lines show the daily change in rank and solid lines the cumulative change over time: $\Delta r(t)= r_{\mathrm {No\text {-}response}}(t)-r_{\mathrm {Hard\ lockdown}}(t)$. Positive values indicate movement *up* the ranking under the policy (greater relative burden; maladaptation), while negative values indicate movement *down* (reduced relative burden; adaptation); the horizontal dashed line marks $\Delta r=0$.

Applied to our case city, The Hague, the UAI reveals that while the Hard lockdown reduced overall infections citywide, it altered the spatial hierarchy of risk (Fig. 7). The Central district, initially having the highest number of infections, saw a sharp decline and moved down the ranking (negative $\overline{\Delta r}^d=-16.89$), reflecting strong adaptation. In contrast, the Outer residential district moved up the ranking (positive $\overline{\Delta r}^d=+10.01$), meaning its position in the absolute infection hierarchy worsened relative to other districts. Although the policy was effective in aggregate, the UAI highlights a form of spatially uneven adaptation: the policy’s benefits were strongest in the city core, while peripheral areas experienced a relative worsening of their rank.

## Discussion

This study examines how uncontextualised, uniform interventions during an epidemic can unintentionally result in maladaptation in heterogeneous urban environments. Our findings reveal a critical emergent pattern: a uniform ’Hard lockdown’ ’flips’ the risk profile of the city. The Central district (Centrum), the original epidemic hotspot due to its high density of amenities and transient visitor population, sees a significant reduction in infections. Conversely, the Outer residential district (Ypenburg), which had fewer infections in the ’No-response’ scenario, becomes the new city hotspot, surpassing the centre in absolute infections.

The primary mechanism for this ’flipped risk’ is the clash of behavioural adaptation with inequitable local service capacity. In the Central district (Centrum), the lockdown is highly effective because it shut down the amenities and workplaces that attracted a large transient population, cutting the virus from its main transmission routes. In both districts, residents adapted their essential travel, stopping ’trip-chaining’ (e.g., shopping near work) and defaulting to their closest local supermarket. This is where the maladaptation occurred. In the Outer residential district (Ypenburg), which has very few supermarkets, this rational, adaptive behaviour inadvertently concentrated the entire district’s population into a handful of essential ’hubs’. These locations, with a low service capacity relative to the population they serve, became new infection hotspots, driving an increase in cases that surpassed the city centre.

Our findings align with research highlighting the heterogeneous spread of viruses in cities^32,33^ and the role of places people visit^19,21^, but add a crucial distinction. Much recent literature has advocated for the ’15-minute city’ as a pandemic-resilient model, focusing on equal access to services^34^ or diversity^35,36^ to reduce infections. Our results challenge this, suggesting that local service capacity may be more critical than mere access during a crisis. While Ypenburg’s residents had access to other supermarkets across the city, their adaptive behaviour, a form of ’threat rigidity’ where individuals revert to the simplest, most habitual response^37^, drove them to the closest option. When local capacity is low, this ’rigid’ individual adaptation becomes maladaptive for the district population.

This directly exposes the pitfalls of uniform policies and raises critical questions of spatial equity^38^. A policy that appears ’equal’ by applying the same rules to all is, in fact, spatially ’inequitable’. It ignores the underlying heterogeneity that dictates how the policy is experienced, amplifying vulnerability in already underserved areas^39,40^. While previous work has noted that crises disproportionately affect vulnerable individuals^41^, our model shows how interventions can create vulnerable districts, underscoring the need for interventions centred on local capacity, not just access^42^.

**Fig. 8:**
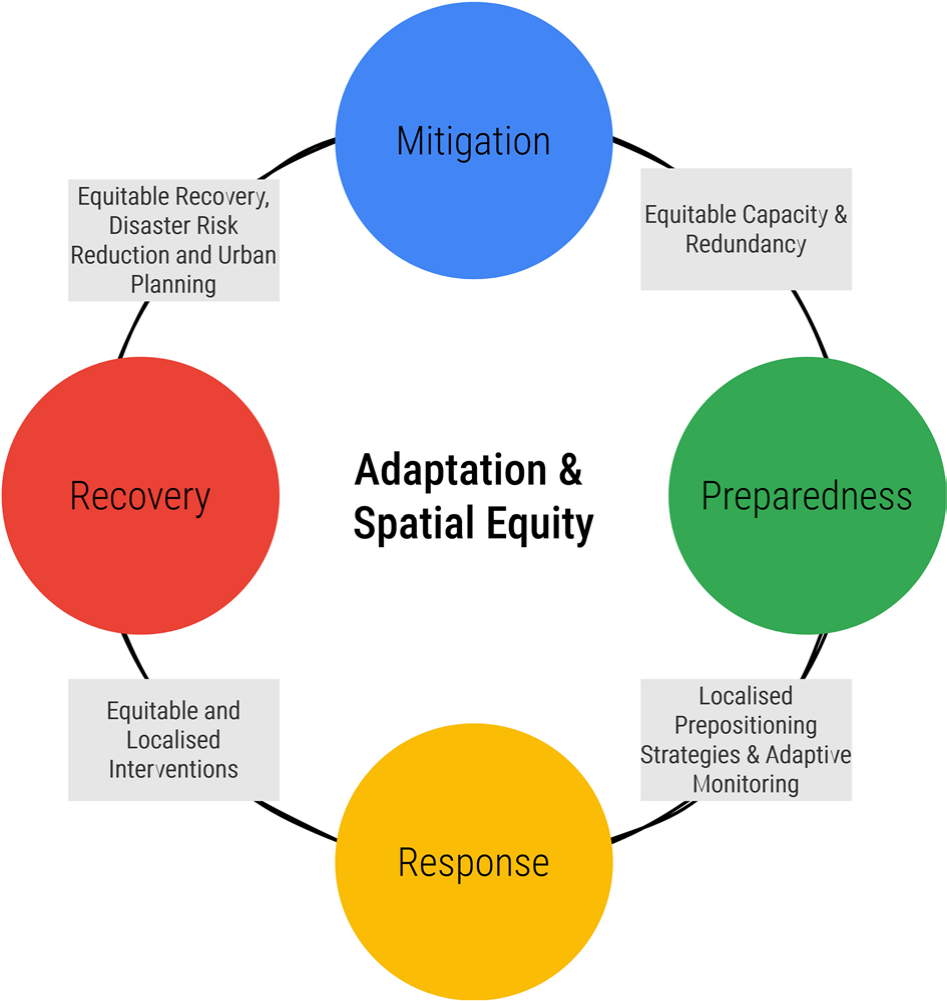
Adaptation and spatial equity-oriented crisis management cycle. The diagram illustrates the four critical stages - Mitigation, Preparedness, Response, and Recovery - each guided by the proposed principles that could foster successful adaptation and promote spatial equity throughout the crisis management process.

To address this, we propose integrating adaptation and principles of spatial equity directly into the crisis management cycle (Fig. 8). This begins with conducting more equitable risk assessments during the Mitigation and Prevention phases, ensuring that all urban populations are considered and their vulnerabilities are not exacerbated. These assessments should identify and address systemic inequalities that often leave marginalised communities disproportionately exposed to risks. During the Preparedness phase, it is crucial to consider the broader social, economic, and environmental contexts that arise from existing urban inequalities. This means developing preparedness plans that are not only technically sound but also socially inclusive, taking into account the unique needs and capacities of different urban neighbourhoods. Incorporating spatial equity principles into the Response phase involves developing flexible strategies that adapt to changing conditions and behavioural responses while ensuring equitable resource allocation. This approach helps prevent the reinforcement of existing inequalities, flipping the risks from one area to another and promoting a more unified and effective crisis response. Finally, the Recovery phase should emphasise lessons learned from various social groups’ behavioural responses and system vulnerabilities, focusing on addressing these disparities to build a more resilient urban environment. Recovery efforts should prioritise rebuilding in ways that enhance long-term resilience for all communities, ensuring that no group is left behind.

It is important to position this research within its methodological context. This study is exploratory: its purpose is not prediction but to build the theoretical and conceptual foundations for understanding urban maladaptation^23,24^. The agent-based model serves as a ’computational laboratory’ fit for this purpose: to identify the underlying mechanisms and emergent patterns that result from complex interactions between policy, behaviour, and urban form. To this end, we deliberately selected a ’most different cases’ design^43^. By comparing two districts with fundamentally different urban characteristics (central vs. peripheral, high vs. low amenity density, transient vs. residential population), we could isolate how and why the same uniform policy produces spatially divergent outcomes.

This exploratory approach necessarily has limitations. First, any model is an abstraction^44^. Therefore, multiple real-life objects and processes are either simplified or aggregated. We limit ourselves to modelling only citizens, their behaviour, and the businesses and services they use. Previous research has demonstrated that these components are critical for understanding the disease’s spread^6,21,30^. However, others also stressed the importance of public transport as the key driver of COVID-19^26^. Second, we focus on the first wave of the pandemic when the residents’ response is rather uniform. Further into the pandemic, we have observed much more complex response patterns, e.g., ’pandemic fatigue’^45,46^. Given the district’s characteristics and the crisis phase, further work can be centred around incorporating other types of adaptive behaviour into the model.

In conclusion, our study demonstrates that uniform, uncontextualised interventions during a pandemic can lead to maladaptation in spatially heterogeneous urban environments, flipping the risk from the urban core to peripheral residential areas. This outcome is not random but a ’predictable’ result of behavioural rigidity interacting with an inequitable distribution of local essential service capacity. These findings, generated from our exploratory approach, can be framed as testable propositions for future research:

- Districts with higher local capacity of essential services demonstrate greater positive adaptation (i.e., risk reduction) to lockdown-style interventions.
- The effectiveness of uniform interventions is fundamentally tied to the primary population of a district (e.g., transient visitors vs. permanent residents).
- Local service capacity, not just spatial access, is a key determinant of intervention effectiveness during a mobility-restricting crisis.

These findings underscore the critical need for adaptive, context-specific, and equity-aware crisis strategies that account for the heterogeneous nature of modern cities. By adopting such strategies, policymakers and crisis managers can build stronger urban resilience against future epidemics and other crises, ensuring more equitable outcomes for everyone.

## Methods

This section starts with a high-level overview of the model. To facilitate its understanding, we follow a common approach in the agent-based modelling studies: Overview, Design concepts, Details (ODD) protocol’s summary. The rest of the section dives deeper into each of the model’s ’building blocks’, and Supplementary Information elaborates on each of them.

### Introduction to the model

The purpose of the model is to evaluate the impact of different interventions on the spread of the COVID-19 virus throughout urban districts. The basic idea is that to understand how an airborne virus spreads, we must simulate human behaviour at the individual level and interactions among people in cities at a high spatio-temporal resolution^20^. Specifically, the model addresses the following question: What is the impact of ’inaction’ (business-as-usual or No-response) and a ’hard lockdown’ (only essential services remain operational) on the two exemplary districts, Central and Outer residential? That said, while focusing on two exemplary districts, we simulate an entire city. Thereby, we avoid all sorts of scaling artefacts. The model comprises four submodels: *person*, *location*, transport, *activity*, and *disease*. The latter consists of *transmission* and *progression* submodels. To consider our model realistic enough for its purpose, we derive patterns in daily citizen activities that form weekly routines, spatially allocated demographics and socio-economics, built environment and places of interest from existing data. We base the epidemiological submodel on peer-reviewed literature and available empirical data.

The model includes the following entities: person and location. A person entity models a resident of a city of a certain age. Each person is associated with a household of one of four types: *single* (one agent), *couple without children* (two agents), *couple with children* (more than two agents) and *single-parent* (two or more agents). Importantly, a household is not an entity but a person’s attribute. People are part of one of 11 social groups (student, pensioner, etc.) based on age and other factors. Each person has a set of activities (schedules) they perform on weekdays and weekends at different locations. Depending on a person’s social group, they have different activities (study, shopping, etc.) to perform and, therefore, various schedules. Assuming all family members stay together, all persons belonging to the same household are placed in a single location of *home* type. Additionally, most persons are also associated with either a *work-* or *studyplace* depending on their social group. Finally, a person has a *disease phase*, e.g. susceptible, exposed, etc., defined in line with the corresponding progression model.

The location entity models residential objects or homes (apartments or houses) and non-residential objects or places of interest (POI). Each object has a pair of coordinates and an area in m2. If it is a POI, it has a category and a subcategory: education category and school subcategory, shopping category and supermarket subcategory, etc. A POI can be indoors or outdoors, such as a park.

As for spatial and temporal scales, the model is spatially and temporally explicit and has high resolution. Persons and locations have a set of latitude and longitude pairs; therefore, the model can report back the outcomes on the building scale (opposite to the district or neighbourhood scale). However, we aggregate the outcomes on the district scale due to the nature of the input data: it is open and anonymised, but district-scale, and, thus, to avoid a false sense of precision.

The model does not have ABM’s timestep concept. It follows the discrete-event formalism, and we can examine a phenomenon or count attributes at a specific moment. For example, instead of checking every 10 minutes the number of citizen-agents in a supermarket, we count the number only when an agent comes or leaves. However, for simplicity’s sake, we collect and aggregate model outcomes by hour. As for the extent, the model represents a single city: The Hague, the Netherlands. By default, there are no incoming and outgoing persons from and to other cities. Thus, the model is a *closed system*.

The most important processes of the model, which are repeated every hour, are the movement of person agents between various locations and their potential infection. Each person has two sets of daily schedules: one for weekdays and one for weekends. A schedule is a combination of routine activities repeatedly executed by an agent over a day. The total number of available activities is 11. For example, sleep at home, travel by public transport, work at a university, shop at the supermarket, travel on foot, and sleep at home. Each activity has a start time, end time and location where it must be executed. The schedules differ by social group. A student agent has a different set of schedules from a pensioner. This process results in the following: multiple agents happen to simultaneously carry out an activity at the same location of a particular floor area. If there is an infectious agent among them, the susceptible agents present may also get infected.

The most important design concept of the model is the spatially and temporally explicit heterogeneous behaviour of agents. Person agents are heterogeneous to an extent and form 11 social groups, each with an associated set of weekday and weekend schedules. The model is spatially explicit, and each agent has a home, might have a work or study place and a schedule, along with the mode of transport and destination of each activity. For example, an agent of a student social group lives in a district called Central and has an activity to do in the evening: meeting with others. The model is designed to first sample the activity’s start time, which in turn depends on how long the previous activities lasted. Next, the model samples the type of location: a bar, restaurant or cafe. Finally, depending on where the agent lives and other attributes (transport), it will sample the exact location and how the agent will get there. Such a design allows us to generate realistic enough dynamics of modern urban areas.

The key process in the model is how the virus spreads. The intuition behind this process is as follows. We integrate the viral load progression within an infected person over time with the likelihood of transmitting the virus to others in closed spaces. The amount of virus (viral load) a person carries changes over time, peaking and then declining, which directly impacts their contagiousness. We translate this understanding into a probability of transmission, factoring in the effectiveness of masks, the duration of exposure between individuals, and adherence to social distancing measures.

### Modelling citizen behaviour via synthetic city

The unique behaviour of each individual citizen collectively forms discernible patterns, often referred to as urban routines^47^. These behaviours are not only temporal (e.g., sleeping at night and working during the day) but also spatial, as people typically perform daily activities, like grocery shopping, in nearby locations, thus forming a *social contact*. Previous research highlighted the importance of social contacts in various places of interest (POI)^21^. Thus, we aim to simulate the virus’s spread dynamics by modelling urban routines and assigning them to agents of different social groups, then situating these activities in specific spaces. The process of modelling urban routines involves five main steps: creating a synthetic population, defining locations, modelling activities, identifying transport choices, and integrating these components in time and space.

### Creating synthetic population

The foundation of any data-driven agent-based model is the synthetic population^48^, forming the *People* submodel. Methods for generating this population vary greatly^49^, with some relying on microdata for accuracy and precision and others using more aggregate or open data due to privacy concerns or data availability, as is the case in the Netherlands. Following the methodology proposed by^50^, we introduce a modified approach utilising open data to generate households and individuals through a stepwise approach informed by aggregate district-level statistics provided by The Hague’s municipal data portal www.denhaagincijfers.nl:

1. **Generate households.** For each district and seed, tabulated counts from district-level census tables are expanded into household records via generate_households, then enriched with socio-economic attributes (e.g., household size, income) by assign_attribute_to_households.
2. **Generate individuals.** Households are decomposed into persons based on their size with generate_individuals, and ages are sampled within age brackets using assign_age_to_individuals.
3. **Assign social group.** Each individual is classified into groups (e.g. pupil, worker, pensioner) by assign_social_groups_to_individuals, reflecting age-dependent participation rates.
4. **Spatial anchoring.** Homes and workplaces are assigned: assign_home_ids attaches home locations and assign_workplaces allocates workplaces based on the number of businesses and institutional establishments data.
5. **Reproducibility.** For each seed, compressed CSVs of households and individuals are saved.

**Fig. 9:**
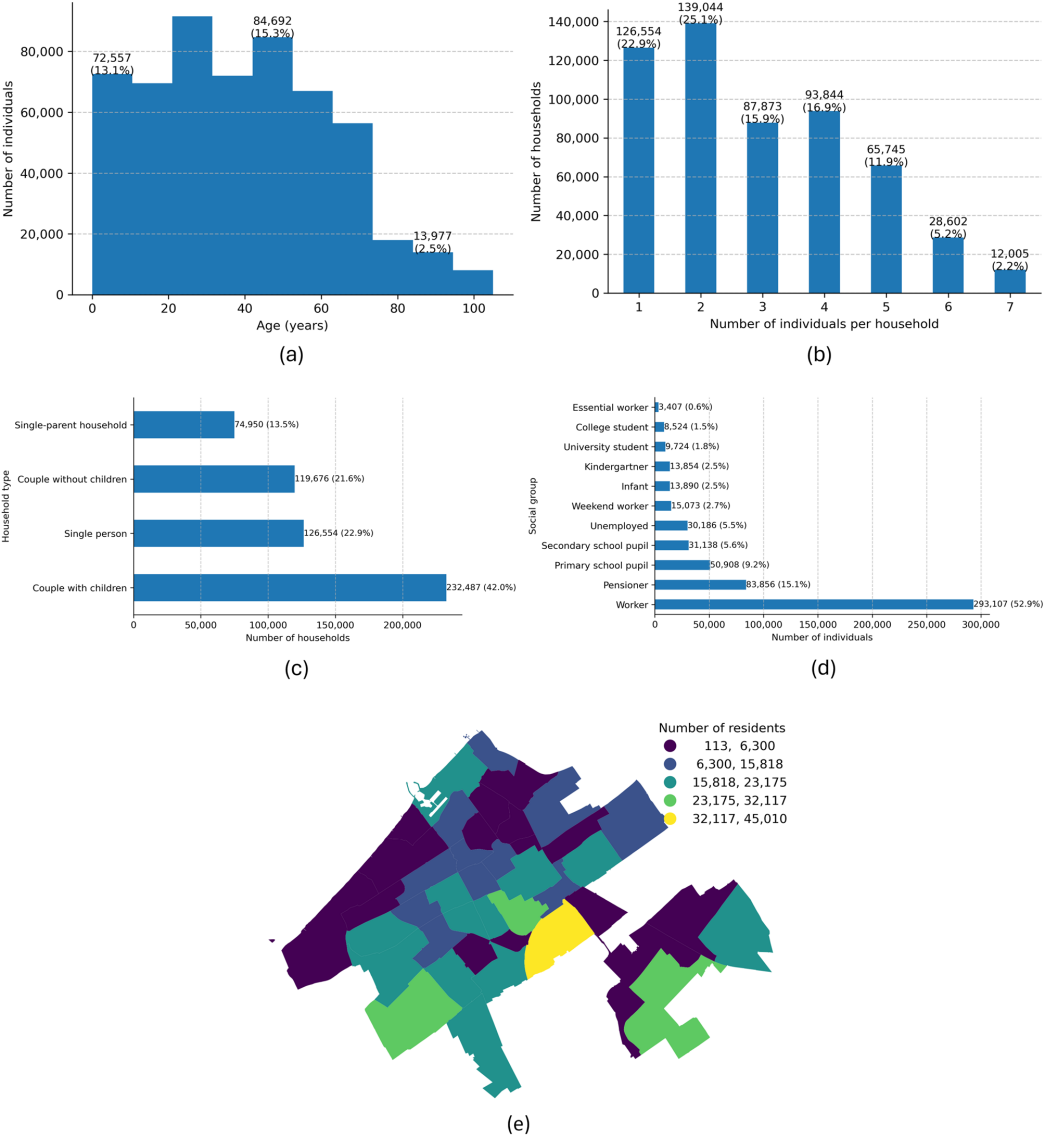
Overview of generated synthetic population for The Hague. (**a**) Age distribution showing the number and share of individuals by age. (**b**) Distribution of household sizes. (**c**) Household type composition. (**d**) Social group structure by age, educational status and/or occupation. (**e**) Number of generated agents-residents in The Hague’s districts.

The resulting synthetic population for The Hague, numbering 553,667 individual agents, is summarised in Fig. 9. This overview validates our generation process against key demographic indicators. The age distribution, shown in panel 9a, reflects a relatively young city, with the largest cohort being adults in their 20–44 years. House composition-wise, the city is dominated by ’smaller units’; while two-person households are the most common type (Fig. 9b, 25.1%), single-person households are nearly as prevalent (22.9%). When categorised by type (Fig. 9c), families with children form the largest group (42.0%), followed by single-person households (22.9%) and couples without children (21.6%). The social group (Fig. 9d) is anchored by a large working population (52.9%) and a significant pensioner group (15.1%), with the remainder composed of students, pupils, and other groups. Finally, panel 9e illustrates the spatial anchoring step, displaying the successful distribution of these synthetic residents across The Hague’s districts, highlighting the population density variations from the city centre to its periphery (for comparison of real and synthetic data see Supplementary Information).

### Defining locations

Businesses and corresponding places of interest (POI) are essential for city life and disease spread^21^, forming the *Locations* submodel. We integrate POI data from OpenStreetMap (OSM) with local urban data from the Municipality of The Hague https://denhaag.incijfers.nl/, detailing the number of businesses by type and their respective employee counts. Previous studies have demonstrated OSM’s utility in crises, particularly in large cities like The Hague^51^.

**Fig. 10:**
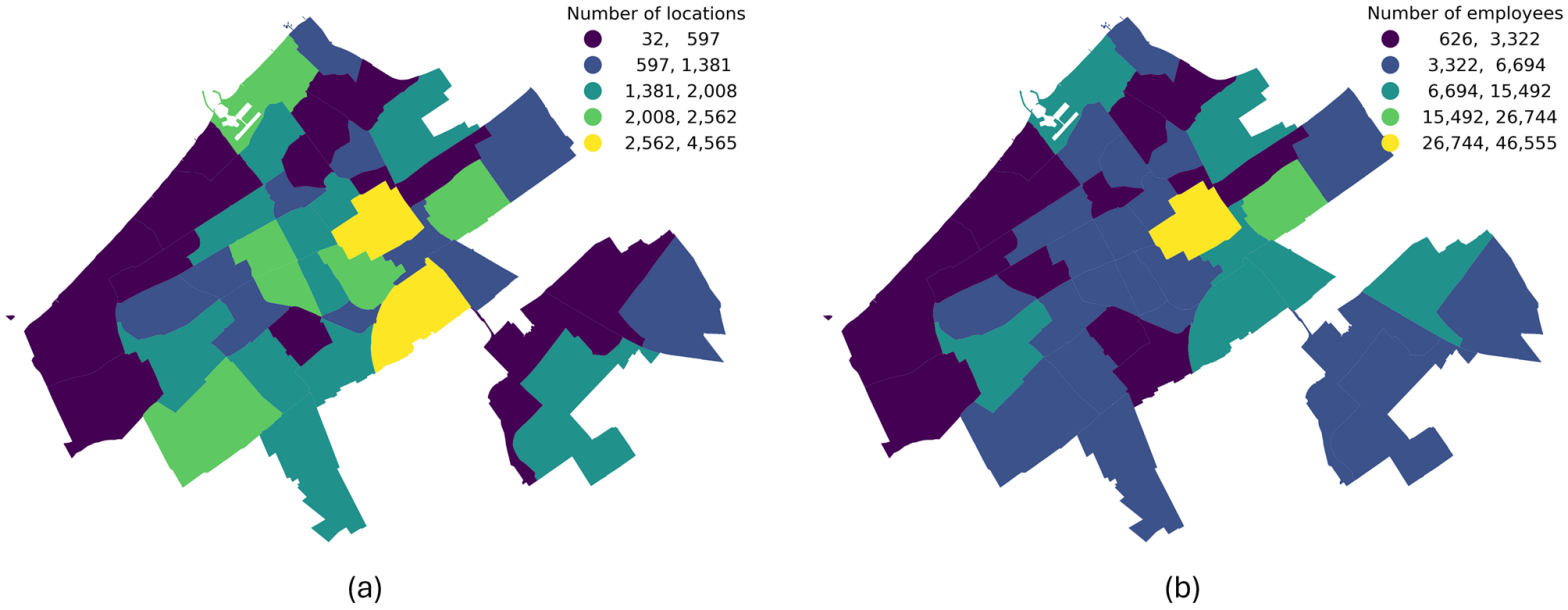
Non-residential locations and employment across The Hague districts. (**a**) Count of places of interest (POIs) per district. (**b**) Number of employees per district.

More specifically, we extract all POIs within The Hague from an OSM data dump dated early January 2020. While OSM’s default schema includes approximately 319 distinct POI types across categories like ’shop’, ’tourism’, and ’leisure’, we aggregate these into 20 broader categories (e.g., ’bars and restaurants’, ’colleges’, see Supplementary Information for a complete overview) for the purposes of this study. Each POI is geolocated with latitude and longitude coordinates and shows significant spatial heterogeneity (Fig. 10a). For example, the city centre contains a higher concentration of supermarkets compared to other districts (Fig. 1). A similar variance is observed in business sizes; after assigning each business to a size category, we sample a specific number of employees from within that category’s range, revealing notable geographic variations (Fig. 10b). Furthermore, we account for sublocations within a single POI, assuming that, for example, a location such as a school comprises multiple classrooms visited by different groups of pupils (for a complete overview of Locations’ statistics, see Supplementary Information).

### Modelling activities

Citizen behaviour is captured through urban routines, which form the *Activities* submodel. We utilise the Time Use Survey (TUS) in the Netherlands^52^, where respondents from different social groups record their activities in 10-minute intervals over a week. This data creates a weekly calendar detailing sleep, work, study and other activities. These schedules inform the activities of agents from each social group, specifying the day and duration of each activity.

**Fig. 11:**
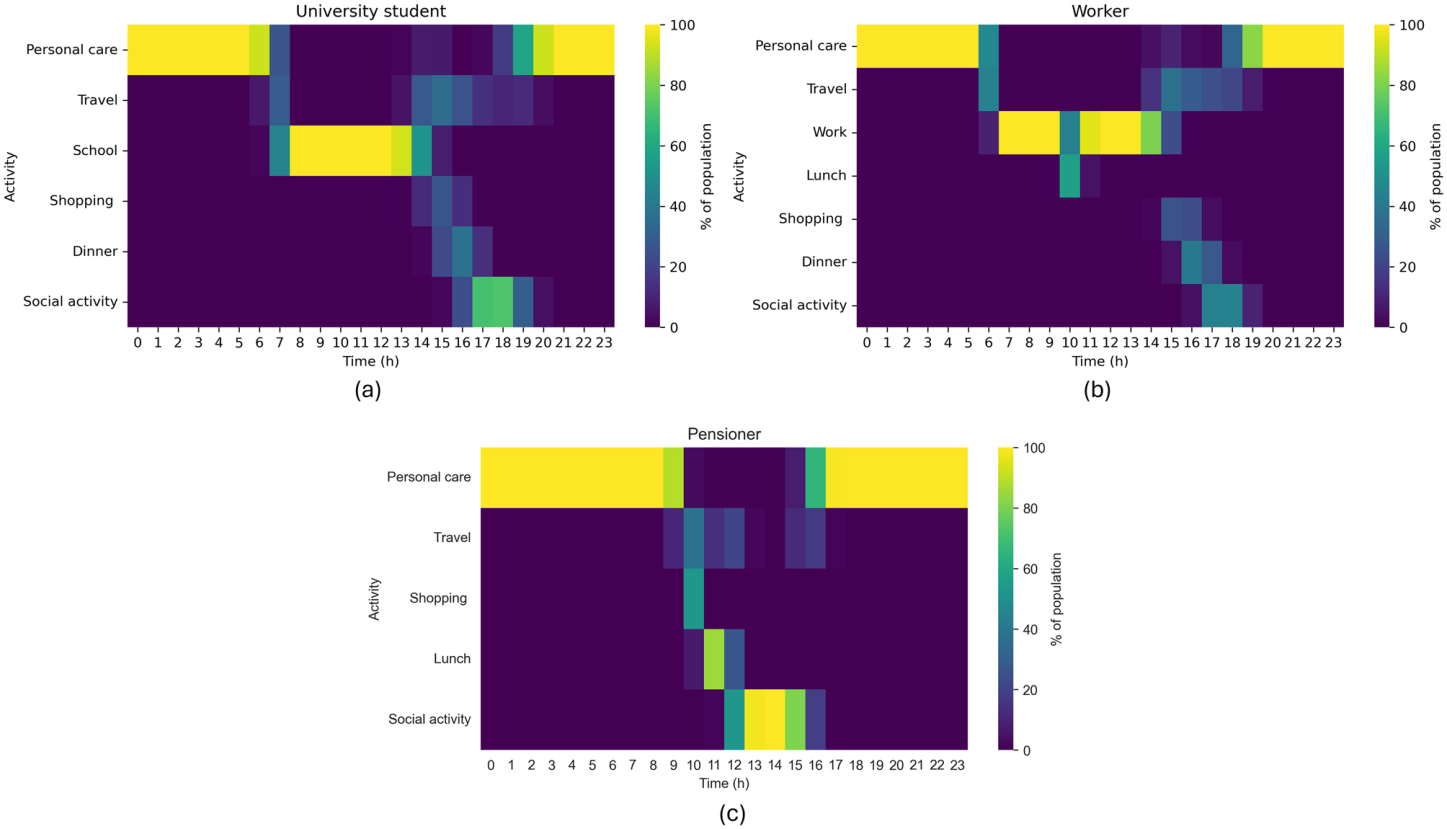
Daily activity schedules (Monday) by social group in the pre-pandemic baseline. Heatmaps show the share of each group engaged in an activity by hour. (**a**) University students. (**b**) Workers. (**c**) Pensioners.

A schedule is a nested data structure defined by seven hierarchical levels: policy, epidemiological state, day of the week, social group, activity, time, and location. We construct these schedules for each of the 10 active social groups (infants are excluded and assumed to remain at home). The schedules capture distinct temporal patterns, differentiating between weekdays and weekends to reflect significant variations in daily routines. Drawing from the work of^52^, we model urban life using nine primary activities: personal care, travel, work or school, lunch, dinner, shopping, and social activities. Each activity is linked to a specific location type; for instance, studying must occur at a school or university (Fig. 11).

The duration of each activity is determined by stochastically sampling start and end times from distributions derived from the TUS data. The choice of location is also partially stochastic. While locations for routine commitments like work or school are sampled once at the simulation’s start and remain fixed, locations for other activities like shopping or socialising are re-sampled for each occurrence. This sampling process is typically guided by proximity; an agent will likely select the closest supermarket, but may choose a random bar or restaurant within a 2.5 km radius.

Finally, this baseline schedule is dynamically overridden by two key factors. First, the active policy can alter behaviour. Under a baseline scenario with no policy intervention, agents follow their normal schedules. However, the Hard lockdown policy restricts activities to essential ones, such as grocery shopping, though essential workers also continue to work. Second, an agent’s epidemiological state, aligned with an extended SEIRD model, can supersede the schedule. Agents who become ’Hospitalised’ or are in the ’ICU’ are removed from the general population and confined to a hospital location. Upon entering an infected state, symptomatic agents-individuals stay home, whereas asymptomatic individuals continue their normal routines (schedules for the rest of the social groups can be found in Supplementary Information).

### Identifying travel patterns

Modelling realistic citizen behaviour relies critically on understanding their travel patterns. In our simulation model, these patterns are essential in connecting the *People*, *Activities*, and *Locations* submodels, and they collectively form the *Transport* submodel. We define a travel pattern as a combination of three core components: the activity an agent intends to perform, the travel mode they choose, and the distance they travel.

To parameterise these patterns with empirical data, we utilise the 2019 Onderweg in Nederland (ODiN) survey, a comprehensive national travel behaviour dataset. For this study, we zoom in on the urban travel patterns within The Hague to derive representative travel distances and population shares.

To align the data with the objectives of our epidemiological model, we performed two key simplifications. First, we aggregated the 14 original activity types found in the ODiN survey into five broader categories relevant to disease spread (e.g., ’Work or school’, ’Shopping’). Second, we collapsed the numerous transport modes into three primary groups: ’Car’, ’Walking/Cycling’, and a ’Rest’ category. This ’Rest’ group includes public transport as well as other miscellaneous modes.

Table 1 summarises the results of this data processing. It details the population share for each of our three simplified transport modes and provides key descriptive statistics for trip distance, broken down by our five aggregated activity types.

**Table 1:** Travel behaviour summary for grocery shopping and other key activities. Left: population share by transport mode; right: trip-distance distribution (km) by activity (mean, SD, min, P25, median, P75, max). ’Rest’ groups public transport and other less-common modes.

Activity	Population share (%)			Trip distance (km)						
	Car	Rest	Walking/Cycling	Mean	SD	Min	P25	Median	P75	Max
Work or school	25.2	42.3	32.5	19.39	29.31	0.1	3.1	7.8	21.5	272.9
Shopping	33.6	19.2	47.2	6.63	14.07	0.1	1.0	2.5	5.9	151.0
Social activities	30.5	16.2	53.3	15.15	28.77	0.1	2.0	5.0	12.0	237.8
Personal care	33.7	28.0	38.3	15.48	29.94	0.1	2.0	5.0	13.0	269.4

### Integrating components

The final and most critical phase of the model’s construction is to integrate these distinct, data-driven submodels - *People*, *Locations*, *Activities*, and *Transport* into a single, coherent simulation of daily urban life. This integration works in two stages: static ’anchoring’ at initialisation and dynamic execution at runtime.

First, we perform the static anchoring of the population. The synthetic agents generated in the *People* submodel are spatially anchored within the built environment defined by the *Locations* submodel. Each household is assigned to a specific residential building within its designated district, establishing a fixed ’home’ location. Likewise, ’workers’ or ’students’ agents are assigned to a fixed workplace or educational institution, respectively, sampled from the available non-residential POIs.

Second, the dynamic integration takes place at runtime as agents execute their daily schedules derived from the *Activities* submodel. While ’home’, ’work’, and ’school’ locations are fixed, other flexible activities (e.g., ’shopping’, ’social activity’) require a dynamic location choice. This process synthesises within the *Activities* and *Transport* submodels. When an agent initiates a flexible activity, the model does not select from a predefined list. Instead, it consults the empirical travel pattern data to sample a travel distance from that activity’s specific distribution. The agent then selects a POI of the correct category (e.g., ’supermarket’) from the *Locations* submodel that is reachable within that sampled distance.

This sequential process - linking People to Locations at initialisation, and then using Activities and Transport data to dynamically guide movement between them ensures that all components are fully integrated. This synthesis generates the realistic spatio-temporal mobility patterns and subsequent social contacts that are essential for simulating disease transmission.

### Disease transmission and progression models

The *Disease* submodel in our agent-based model consists of two primary components: the *transmission* model and the *progression* model. The transmission model captures the process by which an infected agent can transmit the virus to a non-infected agent within a closed space. The progression model describes how the disease progresses in an infected agent based on age and COVID-19-specific factors. Together, these models simulate the spread of COVID-19 within a city.

One of the key challenges in modelling disease transmission is accurately representing how the virus spreads in a closed environment. This task is complicated by numerous uncertainties, including factors related to droplet transmission, types of activities, and environmental conditions. While previous work, such as^53^, has addressed some of these issues, their models are often difficult to scale to the level of an entire city.

To address this, we extend the work of^54^ by incorporating the newly available epidemiological insights (e.g., development of viral load over time, etc.). At the core of our transmission submodel is the infection probability $p_i$ of person *i* (where persons $j \in \{1,...,M_k\}$ are infectious, and persons $i\in \{1,...,N_k\}$ are susceptible), which we define as follows:

$$p_i = 1 - e^{-\frac{\beta \cdot p_B \cdot t_{i,j}}{\sigma _T \cdot A_k} \sum _{j=1}^{M_k} p_j(t_e)}$$

where:

- $M_k$ represents the number of infectious individuals in the *k*-th location,
- $\beta$ is a correction factor for mask wearing and other personal protection measures (range: [0, 1]),
- $p_B$ is the base contagiousness parameter of the virus variant,
- $t_{i,j}$ is the time that person *i* and person *j* spent together in hours,
- $\sigma _T$ is a correction factor for ventilation and social distancing for location type *T* (range: (0, 1]),
- $A_k$ is the area of the *k*-th location in square meters,
- $p_j(t_e)$ is the infectiousness of person *j* at $t_e$ hours since exposure, following a triangular distribution defined by:
  - $t_{e,\text {min}}$: the first time after exposure when a person becomes contagious,
  - $t_{e,\text {mode}}$: the time after exposure when a person is most contagious (peak infectiousness),
  - $t_{e,\text {max}}$: the last time after exposure when a person is contagious,
  where $$p_j(t_e) = {\left\{ \begin{array}{ll} \frac{t_e - t_{e,\text {min}}}{t_{e,\text {mode}} - t_{e,\text {min}}} & \text {if } t_{e,\text {min}} \le t_e < t_{e,\text {mode}} \\ \frac{t_{e,\text {max}} - t_e}{t_{e,\text {max}} - t_{e,\text {mode}}} & \text {if } t_{e,\text {mode}} \le t_e \le t_{e,\text {max}}\\ 0 & \text {otherwise} \end{array}\right. }$$

This *area-based* model simplifies the transmission dynamics by directly using the physical area of locations rather than computing interpersonal distances. It assumes uniform mixing within a (sub)location and models the infection risk as inversely proportional to the available area. The infectiousness over time follows a triangular distribution.

Once an infection event occurs between a susceptible and an infected agent, the susceptible agent may become infected, depending on the probability defined by the transmission model. Following infection, the agent transitions through various states (compartments) as the disease progresses, such as from infected to hospitalised or recovered.

**Fig. 12:**
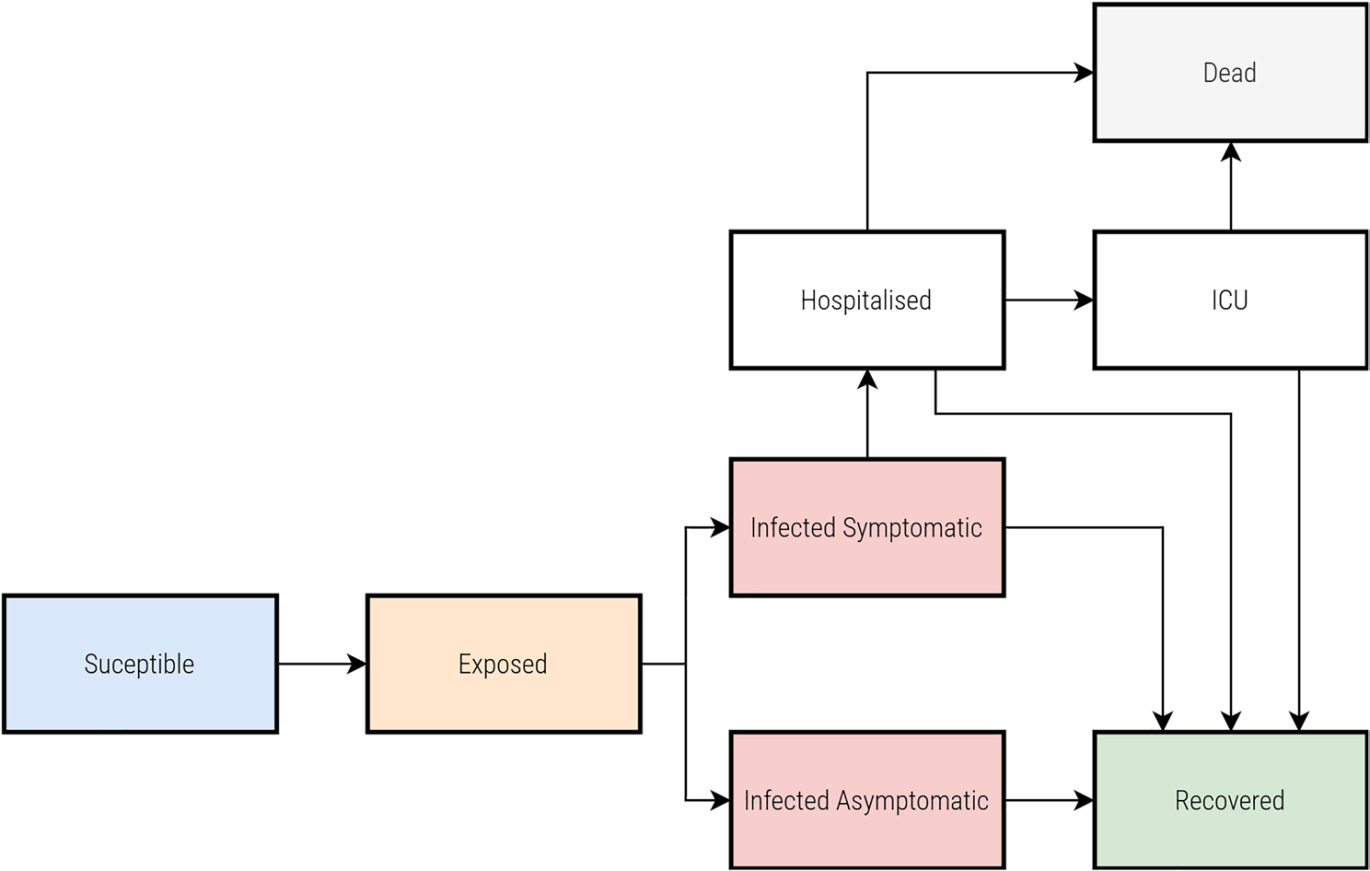
A modified SEIRD model that accounts for asymptomatic infection.

We employ a modified SEIRD (Susceptible-Exposed-Infected-Recovered-Dead) model^55^. This model does not consider reinfection (Fig. 12), which is particularly suited for our study’s focus on the first wave of the pandemic over a short-term period of 90–120 days.

A key feature of our modified SEIRD model is the distinction between symptomatic and asymptomatic infections^56^. This distinction is crucial because symptomatic agents are assumed to alter their behaviour by staying at home, while asymptomatic agents continue their routines, potentially spreading the virus further.

We note that the parameters for both the transmission and progression models are based on the best available data as of June 2020, including works of^56–64^. A key aspect of our methodology, common for large-scale, data-driven agent-based models of this nature, is that we do not perform fine-grained calibration of individual submodels; we prioritise keeping these components as close to the empirical data as possible. Such an approach is especially relevant given the significant uncertainty inherent in modelling human behaviour under an unprecedented crisis like COVID-19.

Consequently, our approach to the epidemiological model is one of validation rather than calibration. We aim to validate the emergent outcomes (i.e., infection dynamics) that arise from the interaction of these data-driven components, rather than calibrating model parameters to fit a specific observed infection curve. This validation-centric approach aligns with the model’s primary purpose: to explore the dynamics of virus spread at an urban scale under different interventions, not to predict the exact number of infections.

Default values and corresponding references of each parameter can be found in the Supplementary Information.

### Experimental setup and output analysis

To evaluate the model, we examine two primary policy scenarios: (1) a ’No-response’ baseline and (2) a ’Hard lockdown’. The baseline scenario simulates ’business-as-usual’, where all urban locations remain open and agents follow their complete, normal activity patterns. To mimic the policy measure which was in place in the Netherlands during the first wave of the pandemic (March 2020), we activate it on day 20 of the simulation model run.

In contrast, the Hard lockdown scenario restricts all activity to essential services, which directly alters agent behaviour in two fundamental ways. First, agents stop activities related to closed amenities. For instance, students no longer attend school, and the time they would have spent elsewhere is now spent at home, increasing potential exposure among household members. Second, the lockdown induces a key behavioural adaptation by changing the spatial pattern of remaining activities. Previously, agents engaged in ’trip-chaining’, such as shopping at a supermarket near their workplace. With work and school travel eliminated, agents now default to shopping at the supermarket closest to their home. This spatial redistribution of essential activities is a critical dynamic in understanding the disease’s spread.

Table 2 details the operational status of each location type for both scenarios. Under the Hard lockdown, non-essential locations (e.g., schools, restaurants, non-food retail, and most workplaces) are closed, while essential services (e.g., supermarkets, pharmacies) and residential accommodations remain open and potential sites of transmission.

Please note two key considerations define the scope of our model: we exclude infections at parks, assuming open spaces and physical distancing mitigate transmission, and we do not model disease spread in hospitals and pharmacies due to their unique environment and specialised safety measures.

**Table 2:** Operational status and infection risk by location type under No-response and Hard lockdown policy scenarios. ’Infection risk’ indicates whether the model allows within-location transmission in that category.

**Location type**	**No-response**	**Hard lockdown**	**Infection risk**
Accommodation	Open	Open	Yes
Bar & restaurant	Open	Closed	Yes
College	Open	Closed	Yes
Fire station	Open	Open	Yes
Food & beverage	Open	Closed	Yes
Healthcare	Open	Closed	Yes
Hospital	Open	Open	No
Kindergarten	Open	Closed	Yes
Mall	Open	Closed	Yes
Park	Open	Closed	No
Pharmacy	Open	Closed	No
Police	Open	Open	Yes
Primary school	Open	Closed	Yes
Recreation	Open	Closed	Yes
Religion	Open	Closed	Yes
Retail	Open	Closed	Yes
Secondary school	Open	Closed	Yes
Supermarket	Open	Open	Yes
University	Open	Closed	Yes
Workplace	Open	Closed	Yes

All simulations are initialised using epidemiological parameters for the Alpha variant of COVID-19, which was prevalent during our March-May 2020 case study period (see Supplementary Information for a complete list of parameters). To initiate the epidemic, we set 100 randomly selected agents to an ’infected’ state. These agents are distributed across the city’s residential areas, representing a realistic early outbreak and ensuring the simulation does not terminate prematurely due to stochastic effects.

Each simulation runs for a maximum of 120 days. We determined this duration through preliminary runs as sufficient to observe whether the epidemic wave infects a significant portion of the city or dies out. While this timeframe is not designed to capture the long-term epidemiological consequences of the virus, it is ideal for focusing on the short-term impacts of the non-pharmaceutical interventions (NPIs) on urban districts. The simulation may conclude earlier than 120 days if the virus is eradicated (i.e., no more infected agents) or if no susceptible agents remain.

The model generates a high-resolution dataset of all transmission events. For this study, we focus on four primary outputs for each infection:

- **Infection location:** The specific POI category where transmission occurred (e.g., home, supermarket, work).
- **Infection district:** The district where the infection location is situated.
- **Residence district:** The home district of the newly infected agent.
- **Infection time:** The simulation day on which the infection occurred.

This analytical setup allows us to differentiate between infections of ’residents’ (where the infection district and residence district are the same) and ’visitors’ (where an agent is infected in a district other than their own). We focus our analysis on two representative districts of The Hague: one central business district, Centrum (’Central’), and one ’Outer residential’ Ypenburg district. To ensure robustness and account for stochastic variability, all reported outcomes represent the average of ten simulation runs, and we include minimum/maximum ranges and error bars where applicable.

### On the validity of the synthetic city

Assessing the validity of any simulation model is a well-known challenge^65,66^, especially when modelling complex human systems and unprecedented scenarios, such as a novel pandemic, for which historical validation data does not exist^67,68^. This challenge is amplified when building a model at the scale of an entire city. Many computational models developed during the COVID-19 pandemic operated at a much smaller scale, for example, simulating a single district or a stylised network, which can introduce scaling artefacts and, more importantly, cannot capture the crucial inter-district dynamics that define urban life^69,70^.

Given these challenges, our take on validity is pragmatic and threefold, rooted in the principles of exploratory modelling for policy analysis^24^. Unlike conventional simulation modelling that aims for precise prediction or optimal solutions, exploratory modelling seeks to understand the range of possible outcomes under deep uncertainty. During the early stages of COVID-19, uncertainty was profound: from the virus’s epidemiological characteristics to human behavioural responses and the long-term impacts of interventions^71,72^. While models that prescribe optimal solutions are useful in stable conditions^73^, our setup focuses on exploring ’what if’ scenarios to identify robust policies and potential vulnerabilities.

Our take on validity supports this exploratory goal and is threefold. First, the model is fundamentally data-driven. As detailed in the preceding sections, it is composed of multiple submodels (e.g., *People*, *Locations*, *Activities*) that are independently parameterised using the best available empirical data (e.g., spatial statistics, time-use and travel surveys). We have demonstrated that each of these components replicates real-world statistics and patterns to a high degree.

Second, we focus on validating the model’s emergent properties. Rather than aiming for point-prediction (e.g., matching the exact number of cases on a specific day), we ensure that the macro-level patterns generated by the simulation, such as mobility flows, activity hotspots, are plausible and resonate with empirical findings reported across various real-world sites.

Finally, a crucial component of our model’s validity is its scale. By simulating the entire city of The Hague, we explicitly avoid the artefacts that arise from modelling a single district in isolation. This comprehensive scale is what allows us to study the heterogeneous impacts of uniform policies and the complex interactions between different urban districts (e.g., a central business district and an outer residential one).

By explicitly modelling the heterogeneity of modern cities, with diverse social groups spread across distinct urban districts, this model serves as a useful tool for estimating the potential district-scale impacts of various interventions. Its primary purpose is not to predict the spread of a virus following an intervention. Instead, it functions as a ’computational laboratory’ to study the interrelation between interventions and their intended, as well as unintended, effects^74^.

### Key assumptions and limitations of the model

Like any simulation model, the one presented in this study is an abstraction of reality and is based on a set of simplifying assumptions^44^. It is important to acknowledge these limitations, as they define the scope and interpretation of our findings.

First, the model’s structure involves several key boundaries. We simulate The Hague as a ’closed system’, meaning we do not account for agent mobility to or from other cities. While this approach avoids scaling artefacts by modelling the entire city, it overlooks the significant commuter and visitor flows that characterise a major urban centre, which could influence infection dynamics. Furthermore, our in-depth analysis focuses on two representative districts. While this ’most different cases’ design is intentional for exploring mechanisms, the findings may not be generalizable to all 44 districts within The Hague, each with its own unique urban and social fabric.

Second, our model of citizen behavior relies on necessary simplifications. The synthetic population is generated from aggregate, district-level open data rather than individual microdata, which inherently limits its granularity. Agent schedules are based on pre-pandemic (2019) time-use and travel surveys. This provides a robust baseline but does not capture any voluntary behavioural changes that may have occurred in the pandemic’s earliest days before a formal lockdown. We also assume a binary response to illness: symptomatic agents stay home, while asymptomatic agents continue their normal routines. This omits more nuanced behaviours, such as partial self-isolation.

Third, the epidemiological and spatial models have specific exclusions. The disease progression model is a modified SEIRD that does not account for reinfection, a simplification considered acceptable for the short 120-day ’first wave’ scenario. Spatially, we excluded transmission in open spaces like parks (assuming low risk) and in hospitals (due to their unique protocols and non-public nature). Moreover, while public transport is implicitly included in the ’Rest’ travel category, it is not explicitly modelled as a distinct, high-contact transmission environment, which other studies have identified as an important driver of spread^26^.

Finally, our experimental design is exploratory. We focus on two starkly different policy scenarios (No-response and Hard lockdown) over a 120-day period. This timeframe is suitable for assessing the short-term impacts of interventions but precludes the analysis of long-term dynamics such as ’pandemic fatigue’^45^, waning immunity, or the impact of subsequent waves and variants.

## Supplementary Information

Below is the link to the electronic supplementary material.

Supplementary material 1

## Data Availability

The datasets generated and analysed during the current study are available at 4TU.ResearchData portal (https://doi.org/10.4121/33c01ff0-d3af-4293-8690-339bbca2bb37).

## Code availability

Current study is available in the Medlabs-HERoS GitHub repository (https://github.com/averbraeck/medlabs-heros). The software used to analyse generated datasets in the current study is available in the urban-maladaptation GitHub repository (https://github.com/miskh/urban-maladaptation).
