# Supplementary material for: On urban maladaptation in times of epidemics
Source: Sci Rep. 2026 Jan 6;16:3171. doi: 10.1038/s41598-025-33158-5 (PMC12830883; doi:10.1038/s41598-025-33158-5)
Supplement: Supplementary file 1 — Supplementary material 1 [file 41598_2025_33158_MOESM1_ESM.pdf]

On Urban Maladaptation in Times of Epidemics

Mikhail Sirenko[1,*], Alexander Verbraeck[1], and Tina Comes[1]

[1]Faculty of Technology, Policy and Management, Delft University of Technology, Delft, the Netherlands
[*]*m.sirenko@tudelft.nl*

Supplementary Information

This section provides additional information on figures and tables from the manuscript's main text. It follows the same structure as the manuscript. Thus, if one is interested in, e.g., figures supplementary to the Results section of the original manuscript, these can be found under the Results.

Results

Figure S1 summarises the city-wide epidemic trajectory under the No-response scenario. We show both the daily count of new infections and the same series expressed as a share of the total population. While our analysis focuses on district comparisons, these trajectories provide context and document between-run uncertainty.

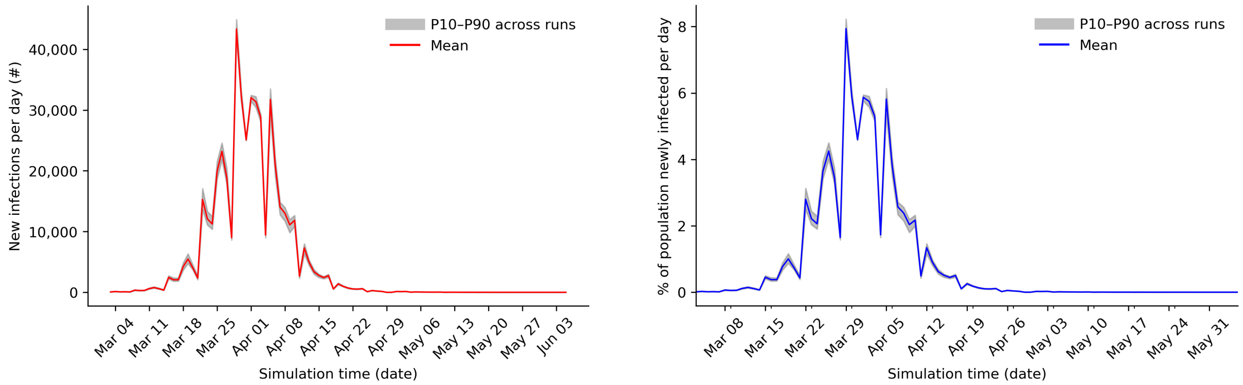

(a) (b)

Figure S1: City-level epidemic dynamics under No-response scenario. (a) Daily new infections. (b) Daily new infections as a share of the total population. Solid line = mean across ten runs; grey band = P10–P90 between-run range. The horizontal axis is the simulation day.

The uncontrolled, city-wide epidemic shown in Figure S1 provides a baseline, but it naturally masks significant variations at the local level. To explore this spatial heterogeneity, Table S1 ranks all 43 urban districts by their mean total infections across the 10 runs.

This ranking immediately reveals the primary hotspots. Centrum (Central) emerges as the most-infected district, accounting for over 8% of all infections in the city. We have also highlighted Ypenburg (Outer residential) (ranked #3), to later observe the change in ranks under the Hard lockdown.

#	District	Infections (mean)	% of total infections (mean)
1	Centrum (Central)	38,772	8.19
2	Laakkwartier en Spoorwijk	33,154	7.00
3	Ypenburg (Outer residential)	26,007	5.49
4	Bouwlust en Vrederust	25,885	5.47
5	Schildersbuurt	23,954	5.06
6	Leidschenveen	19,937	4.21
7	Wateringse Veld	19,537	4.13
8	Morgenstond	18,783	3.97
9	Transvaalkwartier	18,599	3.93
10	Moerwijk	16,032	3.39
11	Rustenburg en Oostbroek	15,543	3.28
12	Bezuidenhout	14,961	3.16
13	Bomen- en Bloemenbuurt	14,587	3.08
14	Valkenboskwartier	14,468	3.05
15	Loosduinen	14,185	3.00
16	Scheveningen	13,720	2.90
17	Leyenburg	12,841	2.71
18	Regentessekwartier	12,094	2.55
19	Waldeck	11,577	2.44
20	Benoordenhout	11,309	2.39
21	Zeeheldenkwartier	10,842	2.29
22	Geuzen- en Statenkwartier	10,422	2.20
23	Mariahoeve en Marlot	9,343	1.97
24	Vruchtenbuurt	7,942	1.68
25	Stationsbuurt	7,870	1.66
26	Belgisch Park	7,227	1.53
27	Duinoord	6,790	1.43
28	Archipelbuurt	5,141	1.09
29	Vogelwijk	4,457	0.94
30	Groente- en Fruitmarkt	4,086	0.86
31	Bohemen en Meer en Bos	3,620	0.76
32	Duindorp	3,543	0.75
33	Hoornwijk	1,974	0.42
34	Willemspark	1,965	0.41
35	Kijkduin en Ockenburgh	1,960	0.41
36	Forepark	1,911	0.40
37	Binckhorst	1,900	0.40
38	Kraayenstein en Vroondaal	1,682	0.36
39	Zorgvliet	1,464	0.31
40	Westbroekpark en Duttendel	1,305	0.28
41	Zuiderpark	1,101	0.23
42	Van Stolkpark en Scheveningse Bosjes	684	0.14
43	Haagse Bos	421	0.09

Table S1: Ranking of urban districts by mean infections across ten runs under No-response. Percentages indicate each district's share of total city infections.

To understand what drives the spread, we must look beyond total infection counts and differentiate who

is getting infected. Our model distinguishes between infections acquired by the district's residents (who live there) and infections acquired by visitors (who work, shop, or socialise there).

Table S2 provides this breakdown for our two focus districts. The model results reveal different infection profiles. Central acts as a major infection hub: visitor-acquired infections (mean of 32,430) vastly outnumber resident infections (mean of 6,342). This is consistent with its function as a high-density, high-mobility commercial and employment centre.

Outer residential shows the precise opposite pattern. Resident infections (mean 19,823) are more than three times higher than visitor infections (mean 6,185). This strongly suggests that transmission in this district is driven primarily by local community and household spread, rather than by external visitors.'

District	Infection type	Mean	Median	Min	Max	Std	P10	P90
Central	Resident	6,342.4	6,345.5	6,258.0	6,423.0	53.98	6,261.6	6,417.6
	Visitor	32,430.2	32,459.5	32,203.0	32,626.0	155.25	32,234.5	32,599.0
Outer residential	Resident	19,822.9	19,878.0	19,259.0	20,296.0	324.24	19,351.7	20,205.1
	Visitor	6,184.6	6,195.0	6,075.0	6,263.0	68.53	6,082.2	6,263.0

Table S2: Key infection metrics for Central and Outer residential districts under No-response, summarised across ten runs (mean, median, min, max, SD, P10, P90).

Figure S2 summarises the city-wide epidemic trajectory under the *Hard lockdown* scenario. When comparing with the *No-response* scenario (see Figure S1), we see a significant decrease in the number of new infections and how the virus dies out.

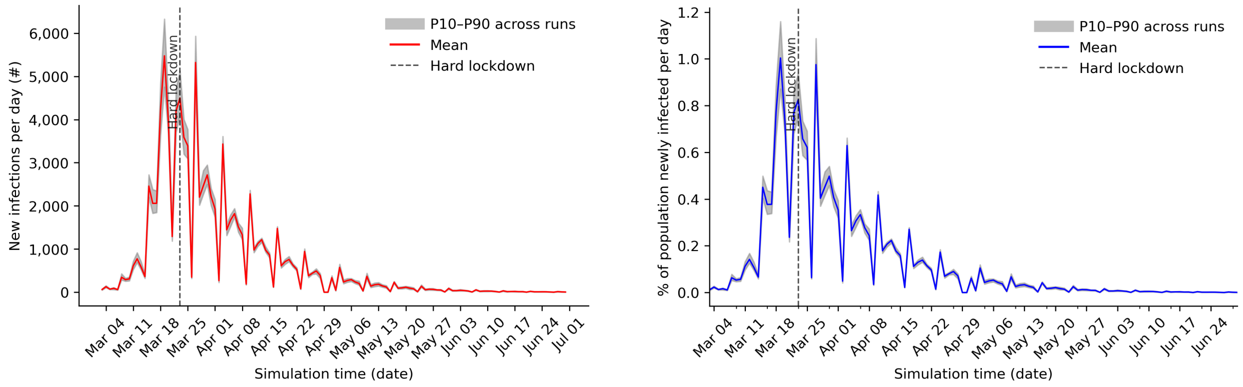

(a) (b)

Figure S2: City-level epidemic dynamics under Hard lockdown scenario. (a) Daily new infections. (b) Daily new infections as a share of the total population. Solid line = mean across ten runs; grey band = P10–P90 between-run range. Vertical dashed line marks policy activation (simulation day 20; 23 March 2020).

As Figure S2 illustrates, the policy intervention is extremely effective at the city level, rapidly reducing the curve. However, this aggregate effect must again be unpacked at the district level.

Table S3 shows the new district infection ranking under the Hard lockdown. A striking 'inversion' has occurred compared to the baseline (Table S1). Centrum (Central), previously the #1 hotspot, has fallen to 10th place. Conversely, Ypenburg (Outer residential) has risen from 3rd place to become the #1 most-infected district in the city, even as its absolute number of infections dropped.

#	District	Infections (mean)	% of total infections (mean)
1	Ypenburg (Outer residential)	11,520	12.82
2	Leidschenveen	9,330	10.39
3	Laakkwartier en Spoorwijk	7,941	8.84
4	Bouwlust en Vrederust	5,861	6.52
5	Wateringse Veld	5,610	6.25
6	Moerwijk	5,302	5.90
7	Morgenstond	4,375	4.87
8	Schildersbuurt	4,155	4.63
9	Transvaalkwartier	2,869	3.19
10	Centrum (Central)	2,520	2.81
11	Rustenburg en Oostbroek	2,204	2.45
12	Loosduinen	2,170	2.42
13	Bomen- en Bloemenbuurt	1,921	2.14
14	Vruchtenbuurt	1,865	2.08
15	Leyenburg	1,826	2.03
16	Waldeck	1,779	1.98
17	Valkenboskwartier	1,761	1.96
18	Scheveningen	1,750	1.95
19	Regentessekwartier	1,582	1.76
20	Geuzen- en Statenkwartier	1,439	1.60
21	Benoordenhout	1,296	1.44
22	Archipelbuurt	1,116	1.24
23	Belgisch Park	1,109	1.23
24	Zeeheldenkwartier	1,027	1.14
25	Mariahoeve en Marlot	1,023	1.14
26	Bezuidenhout	918	1.02
27	Stationsbuurt	905	1.01
28	Duinoord	882	0.98
29	Vogelwijk	672	0.75
30	Groente- en Fruitmarkt	671	0.75
31	Duindorp	649	0.72
32	Hoornwijk	397	0.44
33	Bohemen en Meer en Bos	292	0.33
34	Zuiderpark	194	0.22
35	Forepark	177	0.20
36	Kijkduin en Ockenburgh	151	0.17
37	Willemspark	126	0.14
38	Westbroekpark en Duttendel	124	0.14
39	Zorgvliet	110	0.12
40	Kraayenstein en Vroondaal	75	0.08
41	Binckhorst	64	0.07
42	Van Stolkpark en Scheveningse Bosjes	54	0.06
43	Haagse Bos	19	0.02

Table S3: Ranking of urban districts by mean infections across ten runs under Hard lockdown. Percentages indicate each district's share of total city infections.

The reason for this dramatic re-ranking becomes clear in Table S4, which details the resident/visitor

infection split for the lockdown scenario.

The lockdown policy, which severely restricts mobility, causes visitor infections in Central to collapse by over 94%, from 32,400 to 1,800. In Outer residential, however, while visitor infections also fall, the resident-to-resident transmission remains the dominant mode of infection and is reduced by a more modest 44.5% (from 19,800 to 11,000).

This is the key insight from our model: the Hard lockdown was exceptionally effective at stopping transmission linked to mobility and public/commercial hubs (like Centrum), but it was significantly less effective at stopping the local, community-based transmission that characterises residential areas like Ypenburg.

**District**	**Infection type**	**Mean**	**Median**	**Min**	**Max**	**Std**	**P10**	**P90**
Central	Resident	705.4	713.5	584.0	827.0	86.47	608.3	798.2
	Visitor	1,815.0	1,777.0	1,372.0	2,330.0	271.08	1,515.1	2,051.0
Outer residential	Resident	11,005.3	10,860.5	9,106.0	12,618.0	1,271.01	9,135.7	12,425.4
	Visitor	515.0	507.0	334.0	660.0	100.37	426.7	618.6

Table S4: Key infection metrics for Central and Outer residential under Hard lockdown, summarised across ten runs (mean, median, min, max, SD, P10, P90).

To make the comparison between the two scenarios explicit, Table S5 provides a direct summary of the mean infection counts and the resulting reductions for our two focus districts. This table numerically confirms the differential impact of the lockdown, contrasting the 94.4% reduction in visitor infections in Central with the 44.5% reduction in resident infections in Outer residential.

**District**	**Infection type**	**Mean (NR)**	**Mean (HL)**	**Reduction**	**% Reduction**
Central	Resident	6,342.4	705.4	5,637.0	88.9%
Central	Visitor	32,430.2	1,815.0	30,615.2	94.4%
Central	Total	38,772.6	2,520.4	36,252.2	93.5%
Outer residential	Resident	19,822.9	11,005.3	8,817.6	44.5%
Outer residential	Visitor	6,184.6	515.0	5,669.6	91.7%
Outer residential	Total	26,007.5	11,520.3	14,487.2	55.7%
**Both districts**	Total	**64,780.1**	**14,040.7**	**50,739.4**	**78.3%**

Table S5: Mean infections by district and infection type under No-response (NR) vs Hard lockdown (HL), summarised across ten runs. 'Reduction' is NR−HL; '% Reduction' is $100 \times (1 - \mathrm{HL/NR})$. Totals sum Resident and Visitor; 'All districts' sums across both districts.

Finally, Table S6 shows the data at the city level, providing summary statistics for the entire city across the ten runs for both scenarios. This allows us to quantify the overall impact of the policy and the robustness of the simulation.

	Total infections		% of infected population	
**Statistic**	**No-response**	**Hard lockdown**	**No-response**	**Hard lockdown**
Mean	473,678	89,852	86.81	16.47
Median	473,631	89,252	86.80	16.36
Min	473,076	81,867	86.70	15.00
P10	473,207	83,780	86.73	15.35
P90	474,157	96,924	86.90	17.76
Max	474,453	102,376	86.96	18.76
Std	422	6,043	0.08	1.11
CV%	0.089	6.726	0.089	6.726

Table S6: City-wide comparison of No-response versus Hard lockdown summarised across ten runs. Summary statistics are shown for total infections (count) and infections as a percentage of population; P10/P90 = 10th/90th percentiles; CV% = coefficient of variation.

Methods

The following section provides a more comprehensive overview of the methodology used.

Creating synthetic population

At the core of any data-driven agent-based model lies its primary input data: the synthetic population. This subsection explains the main details on how we modified an approach proposed by (Ge et al., 2014) and the resulting output.

We use the open data portal Den Haag in Cijfers as a source of demographic and socio-economic data about households and individuals (www.denhaag.incijfers.nl). This portal provides district-level data on the number of residents, age distributions, household composition, family size, and income levels. Additionally, it has information about the business locations in The Hague: in which district they are located, their category (e.g., agriculture, industry, etc.), and size in terms of the number of employees. The goal of the approach is to combine these datasets to synthesise a population of individual agents representing the case city residents.

The approach is stochastic and consists of four logical stages, iterating over districts and Monte Carlo seeds. We generate 10 different populations and select the one that best fits the known aggregate statistics. The steps could be described as follows:

Algorithm A1 Synthetic population generator

Require: census tables T_d for districts $d \in D$, seeds S, children bounds $[c_{\min}, c_{\max}]$

for all seed $\in S$ **do**
  for all $d \in D$ **do**
    $H_{\text{syn}} \leftarrow$ GenerateHouseholds($T_d, c_{\min}, c_{\max}$, seed)
    $H_{\text{attr}} \leftarrow$ AssignAttributesToHouseholds(H_{syn}, "income", seed)
    $P_{\text{raw}} \leftarrow$ GenerateIndividuals(H_{attr})
    $P_{\text{age}} \leftarrow$ AssignAgeToIndividuals(P_{raw}, seed)
    $P_{\text{role}} \leftarrow$ AssignSocialGroups(P_{age}, seed)
    $P_{\text{home}} \leftarrow$ AssignHomeIDs(P_{role})
    $P_{\text{work}} \leftarrow$ AssignRWorkplaces(P_{home})
    WriteCSV(P_{work}, "individuals$_d$_$seed${seed}$.csv.gz''$)
  end for
end for

To assess the quality of the proposed approach, we compare the results with the aggregated statistics we used as input data. Figure S3 provides a comparison of the key attributes. Overall, we can conclude that the resulting differences are within acceptable ranges.

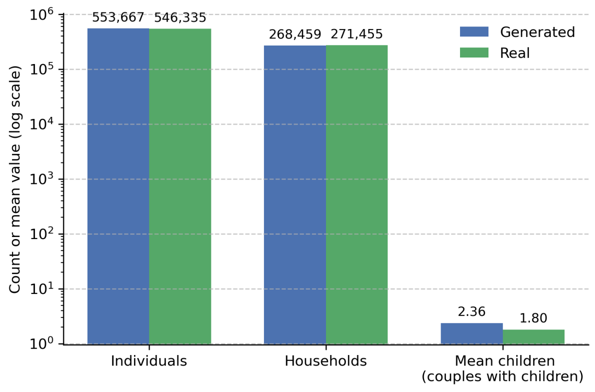

(a)

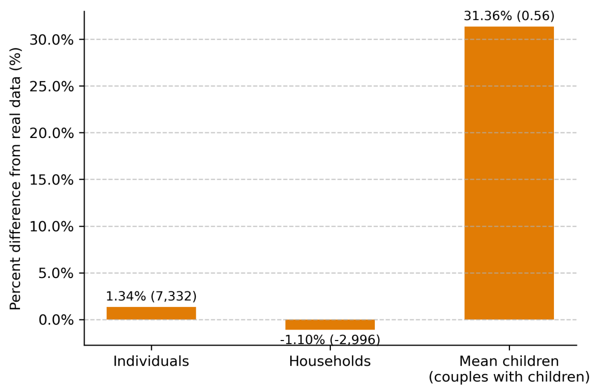

(b)

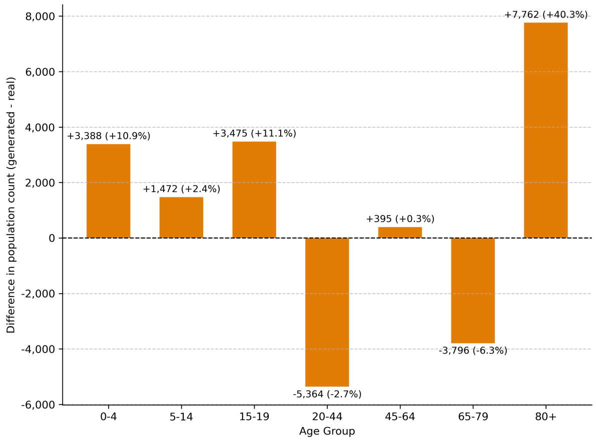

(c)

Figure S3: Validation of the synthetic population against reported aggregates. (a) Generated versus reported totals for individuals and households (log scale) and the mean number of children in couples with children. (b) Percent differences relative to reported values. (c) Age-group population differences (generated – reported).

While this method provides a robust foundation, it is limited by the lack of granular data. Ideally, the synthesis would be informed by individual-level microdata or conditional probabilities to create more realistic attribute connections. Future research could incorporate such data to improve the model's accuracy. Further, the approach incorporates several key assumptions. As a simplifying assumption, each household is assigned to a single home, and we do not model direct interactions between neighbours.

Defining locations

To construct our Locations submodel, we use the official data dump from OpenStreetMap dated the beginning of January 2020. Since the total number of unique places of interest (POIs) is too large for the scope of our study, we aggregate them into 20 relevant categories. Further, we assign each of the POIs a mean area and a number of sublocations given its category. Finally, we combine it with the data on businesses and their sizes (number of employees) per district on 1 January 2020 from the municipal data portal Den Haag in Cijfers https://denhaag.incijfers.nl/. Table S7 represents the result of this approach.

POI category	# of Locations	Total area (m^2)	Mean area (m^2)	Total employees	Mean employees	Total sublocations	Mean sublocations
Accommodation	84,348	8,434,800	100	0	0	0	0
Bar & Restaurant	1,652	330,400	200	12,062	7	1,652	1
College	13	1,300	100	622	47	650	50
Fire Station	6	600	100	376	62	60	10
Food & Beverage	511	51,100	100	1,668	3	511	1
Healthcare	277	27,700	100	3,542	12	831	3
Hospital	13	975	75	5,038	387	8,450	650
Kindergarten	114	5,700	50	2,631	23	684	6
Mall	9	900	100	1,017	113	450	50
Park	21	0	0	0	0	0	0
Pharmacy	63	4,725	75	128	2	63	1
Police	15	450	30	584	38	375	25
Primary School	159	11,925	75	7,691	48	1,590	10
Recreation	244	61,000	250	13,148	53	976	4
Religion	33	8,250	250	223	6	33	1
Retail	2,014	201,400	100	5,629	2	2,014	1
Secondary School	68	5,100	75	2,996	44	2,040	30
Supermarket	133	26,600	200	1,015	7	133	1
University	7	700	100	2,760	394	700	100
Workplace	53,458	1,603,709	29	223,954	4	99,046	1.85

Table S7: Summary of spatially allocated places of interest (POIs) in The Hague Locations submodel (as of 1 January 2020). 'Sublocations' approximate rooms/compartments used for within-POI mixing.

Modelling activities

The key part of the Activities submodel is activity schedules. Figure S4 and Figure S5 display the schedules for the remaining social groups, alongside the ones that were already presented in the main text.

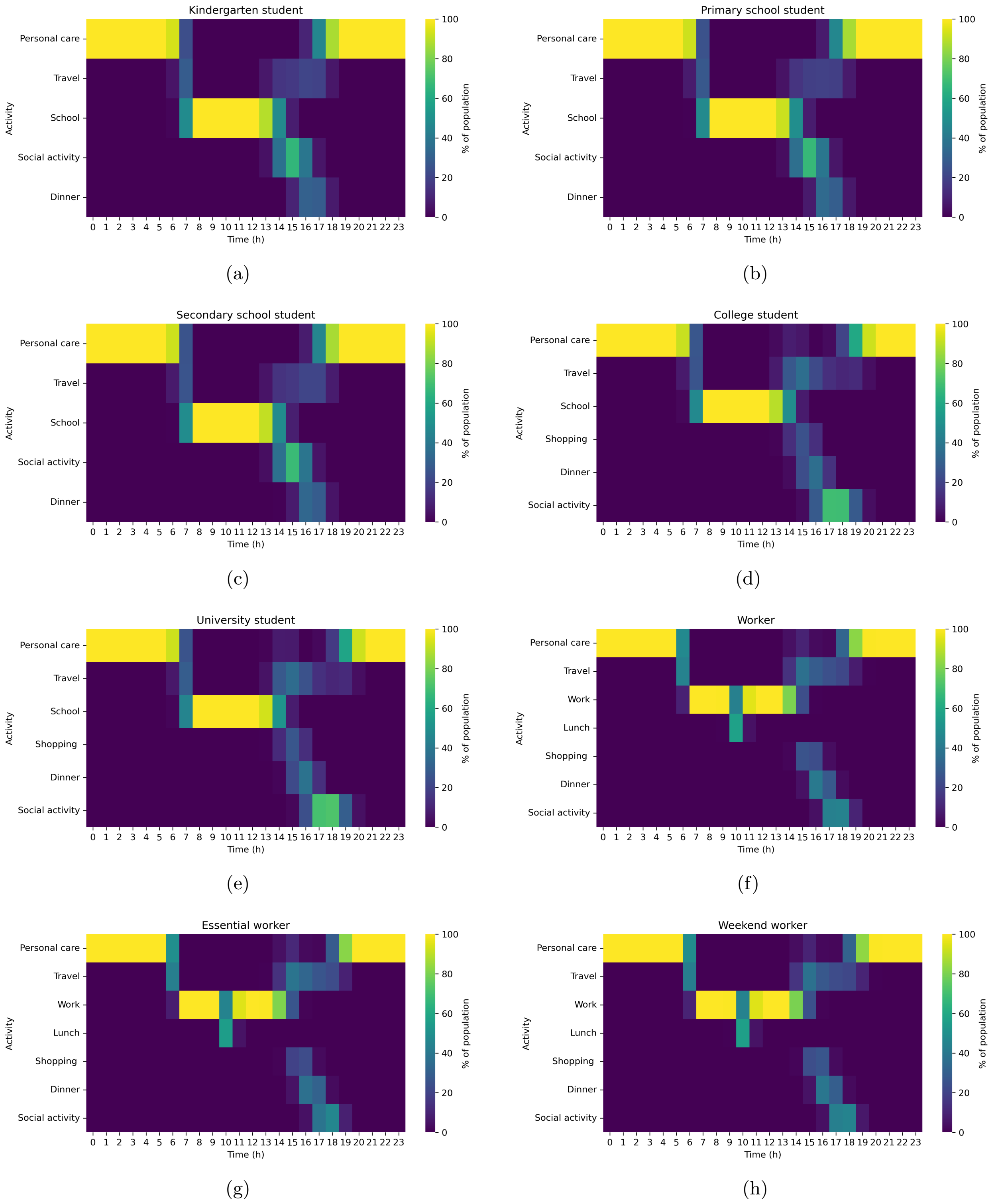

Figure S4: Daily activity schedules (Monday) by social group in the pre-pandemic baseline. Heatmaps show the share of each group engaged in an activity by hour. Panels: (a) kindergarten students; (b) primary school students; (c) secondary school students; (d) college students; (e) university students; (f) workers; (g) essential workers; (h) weekend workers.

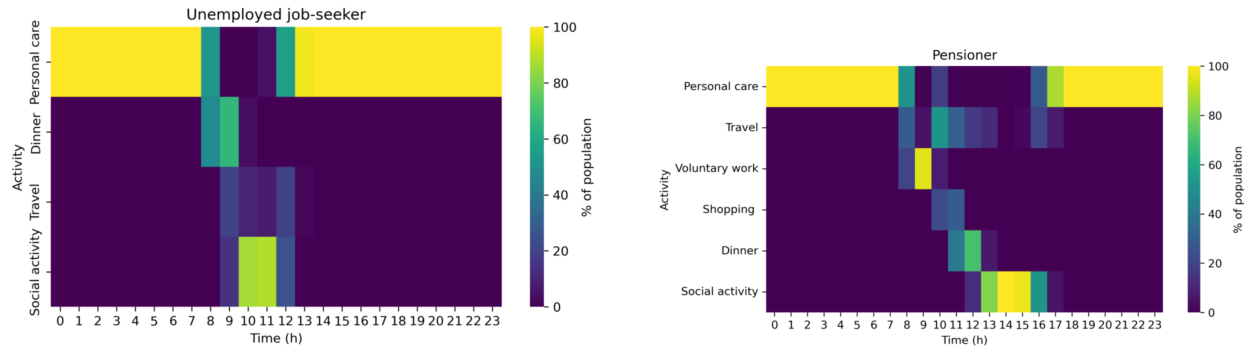

(a) (b)

Figure S5: Daily activity schedules (Monday) by social group in the pre-pandemic baseline. Panels: (a) unemployed job-seekers; (b) pensioners.

Disease transmission and progression models

The core *Disease* component of our model is divided into two interconnected submodels: *Transmission* and *Progression*.

The Transmission submodel defines the probability of an agent becoming infected. This requires defining a set of parameters to determine the infection probability for a susceptible individual based on their exposure to infectious individuals.

The Progression submodel outlines the disease's course after an individual is infected. This component is structured as a modified SEIRD (Susceptible-Exposed-Infected-Recovered-Deceased) model, which we have expanded to include critical clinical states such as 'Hospitalised' and 'ICU'. To operationalise this submodel, we must define three main categories of parameters:

1. **State transitions**: The fraction of individual agents who transition from one disease state to another (e.g., from 'Symptomatic' to 'Hospitalised').
2. **State times**: The duration, or length of time, an individual agent remains in a specific state before moving to the next.
3. **Onset and clinical-presentation parameters**: incubation period and the fraction of infected agents that are symptomatic.

These two submodels are fundamentally linked. An agent's current disease state, as determined by the Progression model, directly influences their level of infectiousness, which in turn is a critical input for the Transmission model.

Transmission model

The Transmission submodel calculates the probability that a susceptible individual i becomes infected while at a specific location k. This probability is a function of their cumulative exposure to all infectious individuals $j \in \{1, .., M_k\}$ present in that same location k. The model is defined by the equation:

$$p_i = 1 - e^{-\frac{\beta \cdot p_B \cdot t_{i,j}}{\sigma_T \cdot A_k} \sum_{j=1}^{M_k} p_j(t_e)}$$

where:

- M_k represents the number of infectious individuals in the k-th location,
- β is a correction factor for mask wearing and other personal protection measures (range: $[0, 1]$),
- p_B is the base contagiousness parameter of the virus variant,
- $t_{i,j}$ is the time that person i and person j spent together in hours,

- σ_T is a correction factor for ventilation and social distancing for location type T (range: $(0, 1]$),
- A_k is the area of the k-th location in square meters,
- $p_j(t_e)$ is the infectiousness of person j at t_e hours since exposure, following a triangular distribution defined by:
  - $t_{e,\text{min}}$: the first time after exposure when a person becomes contagious,
  - $t_{e,\text{mode}}$: the time after exposure when a person is most contagious (peak infectiousness),
  - $t_{e,\text{max}}$: the last time after exposure when a person is contagious,

  where $p_j(t_e) = \begin{cases} \frac{t_e - t_{e,\text{min}}}{t_{e,\text{mode}} - t_{e,\text{min}}} & \text{if } t_{e,\text{min}} \leq t_e < t_{e,\text{mode}} \\ \frac{t_{e,\text{max}} - t_e}{t_{e,\text{max}} - t_{e,\text{mode}}} & \text{if } t_{e,\text{mode}} \leq t_e \leq t_{e,\text{max}} \\ 0 & \text{otherwise} \end{cases}$

To operationalise this transmission model, we must define its static parameters. Other elements, such as the number of infectious people in a location (M_k) or their co-presence time ($t_{i,j}$), are dynamic and calculated by the simulation as it runs. The key static parameters for the transmission model, based on epidemiological literature, are detailed in Table S8:

Symbol	Model variable	Description	Value	Source
p_B	`covidT_area.contagiousness`	Base contagiousness	1.0	Chu et al. (2020)
β	`covidT_area.beta`	Personal protection multiplier (masks)	1.0	Chu et al. (2020)
σ_T	`correctionFactorArea`	Per-location correction factor for ventilation/distancing	1.0	Sun and Zhai (2020); Setti et al. (2020)
$t_{e,\text{min}}$	`covidT_area.t_e_min`	First contagious hour after exposure	2.0 days	He et al. (2020)
$t_{e,\text{mode}}$	`covidT_area.t_e_mode`	Peak infectiousness	3.4 days	He et al. (2020)
$t_{e,\text{max}}$	`covidT_area.t_e_max`	Last contagious hour	9.6 days	He et al. (2020)
—	`calculation_threshold`	Minimum co-presence duration for infection calc.	60 seconds	Model assumption

Table S8: Transmission model static parameters for COVID-19 (Alpha variant).

Please note that while available, neither β nor σ_T was used for obtaining the results of this study; they both are set to 1.

Progression model

For the Progression submodel, we must define the parameters that control the pathway and timeline of the disease. This includes the incubation period, the probability of becoming symptomatic, the age-dependent probabilities of progressing to severe disease, and the time spent in each state.

The tables below detail these parameters, which are based on foundational epidemiological studies from Verity et al. (2020), Ferguson et al. (2020), Linton et al. (2020), Nishiura et al. (2020) and best available open data De Bruin et al. (2020); Geubbels et al. (2023). We define:

1. **State transitions** (S9), which show the age-stratified shares for moving from symptomatic infection to hospitalisation, from hospitalisation to ICU, and from ICU to death.
2. **State times** (S10), which define the duration (modelled with triangular distributions) an individual spends in each state (e.g., 'Asymptomatic', 'Hospitalised', 'ICU') before recovering or progressing.
3. **Onset and clinical-presentation parameters** (S11), which sets the model's incubation period and the overall fraction of infections that become symptomatic.

**From state**	**To state**	**Age range**	**Model variable**	**Value (share, %)**
Infected (symptomatic)	Hospitalised	0–19	covidP.FractionSymptomaticToHospitalized	2.15
		20–29		1.65
		30–39		5.04
		40–49		11.14
		50–59		20.59
		60–69		44.04
		70–79		60.87
		80–89		32.30
		90–100		12.69
Hospitalised	ICU	0–19	covidP.FractionHospitalizedToICU	0.15
		20–29		0.25
		30–39		0.92
		40–49		2.61
		50–59		5.83
		60–69		14.67
		70–79		15.51
		80–89		1.65
		90–100		0.00
Hospitalised	Deceased	All ages	covidP.FractionHospitalizedToDead	0.00
ICU	Deceased	0–49	covidP.FractionICUToDead	0.00
		50–59		1.45
		60–69		8.39
		70–79		39.73
		80–89		63.00
		90–100		67.88

Table S9: Age-specific state-transition shares for COVID-19 (Alpha variant) based on Ferguson et al. (2020).

**From state**	**To state**	**Model variable**	**Modelled values/distribution**
Asymptomatic (infectious)	Recovery	covidP.PeriodAsymptomaticToRecovered	Triangular(12, 16, 20)
Symptomatic (contagious middle)	Hospitalised	covidP.PeriodSymptomaticToHospitalized	Triangular(7, 9, 11)
Symptomatic (contagious middle)	Recovery	covidP.PeriodSymptomaticToRecovered	Triangular(12, 16, 20)
Hospitalised	Recovery	covidP.PeriodHospitalizedToRecovered	Triangular(11, 13, 15)
Hospitalised	Deceased	covidP.PeriodHospitalizedToDead	Triangular(1, 3, 5)

From state	To state	Model variable	Modelled values/distribution
Hospitalised	ICU	covidP.PeriodHospitalizedToICU	Triangular(1, 3, 5)
ICU	Recovery	covidP.PeriodICUToRecovered	Triangular(28, 30, 32)
ICU	Deceased	covidP.PeriodICUToDead	Triangular(2, 4, 6)

Table S10: Clinical progression times by state for COVID-19 (Alpha variant) based on De Bruin et al. (2020) and Geubbels et al. (2023).

Variable	Reported range	Reported mean	Reported distribution	Model variable	Modelled values/distribution	Source
Incubation period (days)	2–14; 4-6	5; 5	Lognormal (best-fit); 95%: 2–14 d	covidP.IncubationPeriod	Triangular(2.5, 3.4, 3.8)	Linton et al. (2020); Liu and Cao (2022)
Share symptomatic (% of infections)	30.8; 40–50	–	Empirical proportion (point estimates across studies)	covidP.FractionSymptomatic	Fixed(0.46)	Nishiura et al. (2020); Ferguson et al. (2020)

Table S11: Onset and clinical-presentation parameters for COVID-19 (Alpha variant). Reference columns report literature values, and model columns show the implemented parameters.

Experimental setup and output analysis

The simulation model is highly configurable, allowing a user to specify an extensive set of parameters. These fall into three categories:

1. **Generic settings**: File paths for input data, simulation duration, and run controls.
2. **Disease parameters**: The COVID-19 transmission and progression parameters detailed in the previous sections.
3. **Policy interventions**: Specific rules or files that modify agent behaviour or location properties.

The user can modify these parameters either through a pop-up window when launching the model or by directly adjusting the corresponding `.properties` files located in the resources folder. The following table lists the complete set of parameters used for this specific experimental setup, including file paths for input data (people, locations, activities), run control settings (e.g., 120-day duration, random seeds), and initial conditions (e.g., 100 initially infected persons at $t = 0$).

Model parameter	Description	Value
generic.InputPath	Base input directory	../../data/thehague
generic.PersonFilePath	People file (relative to InputPath)	people/people.csv.gz
generic.LocationsFilePath	Locations file	locations/locations.csv.gz
generic.LocationTypesFilePath	Location types file	locations/locationtypes.csv

Parameter	Description	Value
generic.ActivityFilePath	Activity patterns	activities/activityschedules_cap.xlsx
generic.ProbRatioFilePath	Prob. ratio / infection modifiers (not used)	epidemiology/infection_rates.csv
generic.osmControlFile	OSM control (interactive animation)	locations/thehague.osm.csv
generic.osmMapFile	OSM map (interactive animation)	locations/haaglanden.osm.pbf
generic.WriteOutput	Write output files?	true
generic.OutputPath	Output directory (created if absent)	./out
generic.RunLength	Simulation duration (days)	120
generic.Seed	RNG seed	111 - 122
generic.PersonDumpIntervalDays	Interval for person dumps (0 = none)	0
policies.NumberInfected	Initially infected at t=0 (persons)	100
policies.MinAgeInfected	Minimum age of initially infected	0
policies.MaxAgeInfected	Maximum age of initially infected	100
policies.LocationPolicyFile	Location policy file	*(blank)*
policies.DiseasePolicyFile	Disease policy file	*(blank)*
generic.diseasePropertiesFile	Disease properties include file	/alpha-area.properties
generic.diseasePropertiesModel	Disease model type	area

Table S12: Simulation configuration: input paths and run control.

References

Chu, D. K., Akl, E. A., Duda, S., Solo, K., Yaacoub, S., Schünemann, H. J., Chu, D. K., Akl, E. A., El-harakeh, A., Bognanni, A., Lotfi, T., Loeb, M., Hajizadeh, A., Bak, A., Izcovich, A., Cuello-Garcia, C. A., Chen, C., Harris, D. J., Borowiack, E., Chamseddine, F., Schünemann, F., Morgano, G. P., Schünemann, G. E. U. M., Chen, G., Zhao, H., Neumann, I., Chan, J., Khabsa, J., Hneiny, L., Harrison, L., Smith, M., Rizk, N., Rossi, P. G., AbiHanna, P., El-khoury, R., Stalteri, R., Baldeh, T., Piggott, T., Zhang, Y., Saad, Z., Khamis, A., Reinap, M., Duda, S., Solo, K., Yaacoub, S., and Schünemann, H. J. (2020). Physical distancing, face masks, and eye protection to prevent person-to-person transmission of SARS-CoV-2 and COVID-19: a systematic review and meta-analysis. *The Lancet*, 395(10242):1973–1987. Publisher: Elsevier.

De Bruin, J., Voorvaart, R., Menger, V., Kocken, I., and Phil, T. (2020). Novel Coronavirus (COVID-19) Cases in The Netherlands.

Ferguson, N., Laydon, d., Nedjati Gilani, g., Imai, N., Ainslie, k., Baguelin, m., Bhatia, s., Boonyasiri, a., Cucunuba Perez, z., Cuomo-Dannenburg, g., Dighe, a., Dorigatti, i., Fu, h., Gaythorpe, k., Green, w., Hamlet, a., Hinsley, w., Okell, l., van Elsland, S., Thompson, h., Verity, r., Volz, e., Wang, h., Wang, Y., Walker, p., Walters, C., Winskill, p., Whittaker, c., Donnelly, c., Riley, s., and Ghani, a. (2020). Report 9: Impact of non-pharmaceutical interventions (NPIs) to reduce COVID19 mortality and healthcare demand. Publisher: Imperial College London.

Ge, Y., Meng, R., Cao, Z., Qiu, X., and Huang, K. (2014). Virtual city: An individual-based digital environment for human mobility and interactive behavior. *SIMULATION*, 90(8):917–935. Publisher: SAGE Publications Ltd STM.

Geubbels, E. L. P. E., Backer, J. A., Bakhshi-Raiez, F., van der Beek, R. F. H. J., van Benthem, B. H. B., van den Boogaard, J., Broekman, E. H., Dongelmans, D. A., Eggink, D., van Gaalen, R. D., van Gageldonk, A., Hahné, S., Hajji, K., Hofhuis, A., van Hoek, A. J., Kooijman, M. N., Kroneman, A., Lodder, W., van Rooijen, M., Roorda, W., Smorenburg, N., Zwagemaker, F., de Keizer, N. F., van Walle, I., de Roda Husman, A. M., Ruijs, C., and van den Hof, S. (2023). The daily updated Dutch national database on COVID-19 epidemiology, vaccination and sewage surveillance. *Scientific Data*, 10(1):469. Publisher: Nature Publishing Group.

He, X., Lau, E. H. Y., Wu, P., Deng, X., Wang, J., Hao, X., Lau, Y. C., Wong, J. Y., Guan, Y., Tan, X., Mo, X., Chen, Y., Liao, B., Chen, W., Hu, F., Zhang, Q., Zhong, M., Wu, Y., Zhao, L., Zhang, F., Cowling, B. J., Li, F., and Leung, G. M. (2020). Temporal dynamics in viral shedding and transmissibility of COVID-19. *Nature Medicine*, 26(5):672–675. Publisher: Nature Publishing Group.

Linton, N. M., Kobayashi, T., Yang, Y., Hayashi, K., Akhmetzhanov, A. R., Jung, S.-M., Yuan, B., Kinoshita, R., and Nishiura, H. (2020). Incubation Period and Other Epidemiological Characteristics of 2019 Novel Coronavirus Infections with Right Truncation: A Statistical Analysis of Publicly Available Case Data. *Journal of Clinical Medicine*, 9(2):538.

Liu, Q. and Cao, L. (2022). Modeling time evolving COVID-19 uncertainties with density dependent asymptomatic infections and social reinforcement. *Scientific Reports*, 12(1):5891.

Nishiura, H., Kobayashi, T., Miyama, T., Suzuki, A., Jung, S.-m., Hayashi, K., Kinoshita, R., Yang, Y., Yuan, B., Akhmetzhanov, A. R., and Linton, N. M. (2020). Estimation of the asymptomatic ratio of novel coronavirus infections (COVID-19). *International Journal of Infectious Diseases*, 94:154–155.

Setti, L., Passarini, F., De Gennaro, G., Barbieri, P., Perrone, M. G., Borelli, M., Palmisani, J., Di Gilio, A., Piscitelli, P., and Miani, A. (2020). Airborne Transmission Route of COVID-19: Why 2 Meters/6 Feet of Inter-Personal Distance Could Not Be Enough. *International Journal of Environmental Research and Public Health*, 17(8):2932.

Sun, C. and Zhai, Z. (2020). The efficacy of social distance and ventilation effectiveness in preventing COVID-19 transmission. *Sustainable Cities and Society*, 62:102390.

Verity, R., Okell, L. C., Dorigatti, I., Winskill, P., Whittaker, C., Imai, N., Cuomo-Dannenburg, G., Thompson, H., Walker, P. G. T., Fu, H., Dighe, A., Griffin, J. T., Baguelin, M., Bhatia, S., Boonyasiri, A., Cori, A., Cucunubá, Z., FitzJohn, R., Gaythorpe, K., Green, W., Hamlet, A., Hinsley, W., Laydon, D., Nedjati-Gilani, G., Riley, S., van Elsland, S., Volz, E., Wang, H., Wang, Y., Xi, X., Donnelly, C. A., Ghani, A. C., and Ferguson, N. M. (2020). Estimates of the severity of coronavirus disease 2019: a model-based analysis. *The Lancet. Infectious Diseases*, 20(6):669–677.
